# Compendium of 5810 genomes of sheep and goat gut microbiomes provides new insights into the glycan and mucin utilization

**DOI:** 10.1186/s40168-024-01806-z

**Published:** 2024-06-06

**Authors:** Ke Zhang, Chong He, Lei Wang, Langda Suo, Mengmeng Guo, Jiazhong Guo, Ting Zhang, Yangbin Xu, Yu Lei, Gongwei Liu, Quan Qian, Yunrui Mao, Peter Kalds, Yujiang Wu, Awang Cuoji, Yuxin Yang, Daniel Brugger, Shangquan Gan, Meili Wang, Xiaolong Wang, Fangqing Zhao, Yulin Chen

**Affiliations:** 1 International Joint Agriculture Research Center for Animal Bio-Breeding, Ministry of Agriculture and Rural Affairs/Key Laboratory of Animal Genetics, Breeding and Reproduction of Shaanxi Province, College of Animal Science and Technology, Northwest A&F University, Yangling, 712100 China; 2 College of Information Engineering, Northwest A&F University, Yangling, 712100 China; 3 Plateau Livestock Genetic Resources Protection and Innovative Utilization Key Laboratory of Qinghai Province, Key Laboratory of Animal Genetics and Breeding On Tibetan Plateau, Ministry of Agriculture and Rural Affairs, Qinghai Academy of Animal and Veterinary Medicine, Qinghai University, Xining, 810016 China; 4 Institute of Animal Sciences, Tibet Academy of Agricultural and Animal Husbandry Sciences, Lhasa, 850009 China; 5 Key Laboratory of Animal Genetics and Breeding On Tibetan Plateau, Ministry of Agriculture and Rural Affairs, Lhasa, 850009 China; 6 College of Animal Engineering, Yangling Vocational and Technical College, Yangling, 712100 China; 7 College of Animal Science and Technology, Sichuan Agricultural University, Chengdu, 611100 China; 8 Institute of Animal Nutrition and Dietetics, Vetsuisse-Faculty, University of Zurich, 8057 Zurich, Switzerland; 9 College of Coastal Agricultural Sciences, Guangdong Ocean University, Zhanjiang, 524088 China; 10 Key Laboratory of Livestock Biology, Northwest A&F University, Yangling, 712100 China; 11 School of Future Technology On Bio-Breeding, Northwest A&F University, Yangling, 712100 China; 12 Computational Genomics Lab, Beijing Institutes of Life Science, Chinese Academy of Sciences, Beijing, 102206 China

## Abstract

**Background:**

Ruminant gut microbiota are critical in ecological adaptation, evolution, and nutrition utilization because it regulates energy metabolism, promotes nutrient absorption, and improves immune function. To study the functional roles of key gut microbiota in sheep and goats, it is essential to construct reference microbial gene catalogs and high-quality microbial genomes database.

**Results:**

A total of 320 fecal samples were collected from 21 different sheep and goat breeds, originating from 32 distinct farms. Metagenomic deep sequencing and binning assembly were utilized to construct a comprehensive microbial genome information database for the gut microbiota. We successfully generated the largest reference gene catalogs for gut microbiota in sheep and goats, containing over 162 million and 82 million nonredundant predicted genes, respectively, with 49 million shared nonredundant predicted genes and 1138 shared species. We found that the rearing environment has a greater impact on microbial composition and function than the host’s species effect. Through subsequent assembly, we obtained 5810 medium- and high-quality metagenome-assembled genomes (MAGs), out of which 2661 were yet unidentified species. Among these MAGs, we identified 91 bacterial taxa that specifically colonize the sheep gut, which encode polysaccharide utilization loci for glycan and mucin degradation.

**Conclusions:**

By shedding light on the co-symbiotic microbial communities in the gut of small ruminants, our study significantly enhances the understanding of their nutrient degradation and disease susceptibility. Our findings emphasize the vast potential of untapped resources in functional bacterial species within ruminants, further expanding our knowledge of how the ruminant gut microbiota recognizes and processes glycan and mucins.

Video Abstract

**Supplementary Information:**

The online version contains supplementary material available at 10.1186/s40168-024-01806-z.

## Background

Sheep (*Ovis aries*) and goats (*Capra hircus*), as globally significant domestic livestock species, have gradually developed unique physiological and behavioral traits that allowed them to adapt to various ecological environments through prolonged natural selection and artificial domestication [1, 2]. Sheep and goats have been widely distributed across different global regions and climatic conditions, demonstrating considerable adaptive survival. Additionally, their evolutionary trajectory and metabolic differences are becoming key focal points in biological research. The ecological niche differences, shaped by the evolutionary habits and environments during their domestication, contributed to the emergence of distinct genomic [3], behavioral [4], and metabolic adaptations [5] between the two species. However, the adaptive capacity of animal species relies not only on the host genome but also on the extensive genetic reservoir of the microbiome [6]. The gut microbiota, as the “second genome” of the host, plays a crucial role in nutrient metabolism [7], immune response [8], host physiology [9], and evolution [10]. Clearly, a comprehensive and in-depth understanding of the differences in the composition and function of the gut microbiota in sheep and goats is essential for a profound insight into the adaptability of these two species during the evolutionary process.

Microbial gene catalogues can serve as a reference for the standardized analysis of microbes across samples and studies [11]. Therefore, sequence reads have been aligned to gene catalogues, allowing researchers to rapidly determine the functional capacity of the gut microbiome [12]. Several gene catalogs for specific ecosystems are being constructed using genomic data, including humans [13–15], mice [16, 17], pigs [18, 19], dogs [20], chickens [21], and cows [22, 23]. However, the lack of specific microbial gene catalogues for sheep and goats is hampering the ability of researchers to screen the gut microbiota of these species for more effective functional strains. Therefore, there is an urgent need for the development of dedicated microbial gene catalogs for sheep and goats. Additionally, with advancements in shotgun metagenomic sequencing and the progress of metagenome-assembled genome (MAG) assembly algorithms, numerous studies aim to reconstruct microbial genomes to better capture the diversity of microorganisms in the gut and other body environments [24, 25]. These studies contribute to the construction of genomes for uncultivated microorganisms, providing crucial foundational data for a more in-depth investigation into the functional aspects of these microorganisms. Previous research has revealed spatial associations between microbial composition, function, and physiological adaptations in different segments of the ruminant gastrointestinal tract using metagenomic data from gastrointestinal samples of seven ruminant species (dairy cattle, water buffalo, yak, goat, sheep, deer, water deer), resulting in the assembly of 10,373 MAGs [26]. However, the existing catalog inadequately represents the microbial diversity of sheep and goats, as it only includes samples from five sheep and six goat gastrointestinal microbial sources [26]. Therefore, there is an urgent need to incorporate a more diverse range of breeds, different rearing environments, and a greater number of sheep and goat samples to construct a high-quality microbial genome library. The metagenome-assembled genome approach will allow a comprehensive analysis of the functional potential of uncultivated microorganisms in the gut of sheep and goats. It will also contribute to a deeper understanding of the unknown role of the sheep and goat gut microbiota in environmental adaptation in different habitats.

Despite the progress in understanding the functional interactions between rumen microbes and their hosts [27], significant gaps persist in our knowledge of gut microbe functions in ruminants. The lack of functional resolution of gut microbial genomes in sheep and goats exemplifies this knowledge gap, hindering our ability to comprehend precise microbial functions in these animals and impeding the development of targeted nutritional interventions. It is increasingly evident that microbes can symbiotically coexist in the gut, and there is a synergistic relationship between the structural diversity of intestinal mucin glycoproteins and the enzyme repertoire of the gut microbiome [28]. Mucin glycoproteins serve as crucial driving factors for the composition and function of the gut microbiota. In turn, the mucosal microbial community impact mucin composition, thickness, immune response, and metabolic health [29, 30]. Therefore, deep analysis of the potential of uncultivated microbes in sheep and goats to encode enzymes capable of degrading complex mucin glycan chains, especially those encoding endo-β-N-acetylgalactosaminidase, fucosidase, N-acetyl-β-hexosaminidases, β-galactosidases, and sialidases [31], is crucial for understanding the interactions between the gut microbiome and host glycans. Our study will provide important microbial resource data for future studies on how microbes regulate intestinal epithelial function and immune responses to pathogens.

In this study, our objective is to construct a gut microbial gene catalog of sheep and goats by utilizing metagenomic assembly technologies, to build a high-quality microbial genome library for sheep and goats, and to elucidate the commonalities and heterogeneities in the composition and function of their gut microbiota between both species. Additionally, we seek to uncover the response mechanisms of gut microbiota composition to different rearing conditions. Through genomic library data, we identified strains with the potential to encode enzymes capable of degrading complex mucin glycan chains. We collected 210 sheep and 110 goat fecal samples from 32 farms representing 21 breeds and conducted in-depth shotgun metagenomic sequencing, providing a comprehensive functional landscape of the sheep and goat gut microbiota. Our study proposes a large-scale annotated bacterial genome database, highlighting the potential of these functional strains as untapped resources.

## Methods

### Animals, sample collection, and transportation

**Fig. 1:**
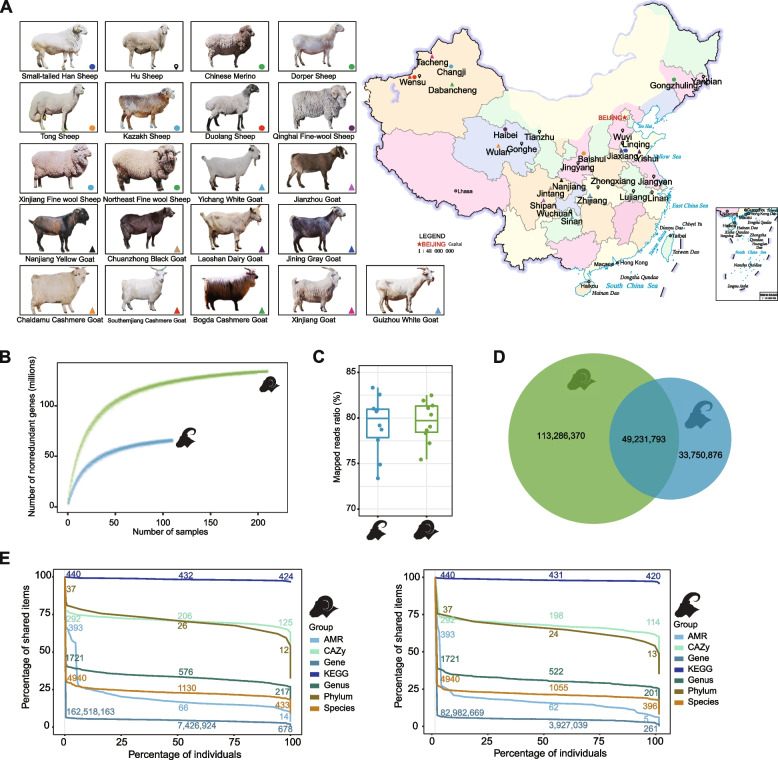
Sheep and goat gut microbial reference gene catalogs. **A** This panel features a montage of the sheep and goat breeds described in this study, each identified on a map using distinct shapes and colors. Circles represent sheep breeds, while triangles indicate goat breeds. A distinct symbol was used to label this region to account for the greater number of samples collected from Hu sheep. **B** The cumulative curve for the NR gene counts concerning sample size. **C** Based on previously published data, the mapping rate of Tibetan goats and wild-blue sheep gut microbiota data to SMGC and GMGC. **D** The numbers of NR genes unique to sheep or goats and those that are shared between the two groups in their gut microbiota gene sets. **E** The proportions of shared bacterial taxonomic and functional items, including phylum, genus, species, KEGG orthologs, CAZy families, resistance genes, and NR genes, across different sample proportions. The *y*-axis and *x*-axis represent shared items and sample percentages, respectively. The figure shows the number and percentage of each shared item at 0%, 50%, and 100% sample proportions

Fresh feces samples were collected by abdominal massage from the rectums of 110 goats and 210 sheep of different breeds and diets from 32 farms (Additional file 2: Table S1). All fecal samples were immediately frozen in liquid nitrogen following rectal sampling. Subsequently, they were transported to the laboratory using dry ice and stored at − 80 °C in the laboratory freezer for future use. Figure 1A depicts the sample collection area. The samples used in this study were collected between April and June 2019. Furthermore, within 6 months of sample collection, all sheep and goats were healthy and were not administered probiotics or antibiotics. In this study, the sheep and goats under grazing conditions refer to animals that, after birth, roam freely in natural grasslands, solely relying on natural foraging without any supplementary of concentrate feed. On the other hand, the sheep and goats under drylot conditions are raised intensively in livestock pens, receiving daily supplementation of concentrate feed in a controlled feeding system. All animal experimental procedures were approved by the Institutional Animal Care and Use Committee of Northwest A&F University (permit number: 20190306001).

### DNA extraction, library construction, and metagenomic sequencing

The cetyltrimethylammonium bromide (CTAB) method was used to extract DNA, according to a previously published protocol [32]. The DNA concentration and purity were determined using a NanoDrop 2000 UV-VI spectrophotometer (Thermo Scientific, Wilmington, DE, USA). The quality of the extracted DNA was assessed using 1% agarose gel electrophoresis. The DNA was stored at − 80 °C until further processing. Overall, 320 extracted DNA samples were fragmented to an average size of approximately 350 bp using Covaris M220 (Gene Company Limited, China) for paired-end (PE) library construction. A TruSeq DNA sample prep kit was used to construct the PE library according to the instructions of the manufacturer. PE sequencing was performed using a NovaSeq 6000 platform according to the instructions of the manufacturer (Illumina, San Diego, CA, USA). Metagenomic sequencing was performed by Microeco Tech Co. Ltd. (Shenzhen, China). Adapter sequences were removed from the 3′ and 5′ ends of the paired-end Illumina reads using Trimmomatic (V1.1) [33] with ILLUMINACLIP: adapters_path:2:30:10 SLIDINGWINDOW:4:20 MINLEN:50. Low-quality reads (length < 50 bp, quality values < 20, or containing N bases) were removed using the Sickle software (v.1.33). Bowtie 2 (V2.3.4.1 –very sensitive) [34] was used to align the reads to the sheep (GCA_000298735.1) and goat reference genomes (GCA_001704415.1), and any hits associated with the reads and their matched reads were removed.

### Metagenome assembly

Metagenome assembly was performed using MEGAHIT (v1.1.3) [35] with the following options: k-list 21, 29, 39, 59, 79, 99, 119, and 141 –min-contig-len 500. Bowtie 2 (v2.3.4.1 –end-to-end, -sensitive) [34] was used to compare clean data to the assembled contigs from each sample to obtain unused PE reads. The unused reads of each sample were merged, and MEGAHIT (v1.1.3) [35] was used for mixed assembly to obtain assembly contigs using the same option. Fragments of < 500 bp were filtered out of contigs obtained by a single sample and mixed assembly, and statistical analysis and subsequent gene prediction were performed.

### Gene catalog construction

We obtained 2.6 Tb of high-quality data from goat samples (an average of 23.60 Gb per sample) and 5.2 Tb of high-quality data (an average of 25.00 Gb per sheep sample) from sheep samples. To construct two gut microbial gene catalogs of sheep and goats, we assembled the Illumina reads from each sample into longer contigs with prodigal (v 2.6.3, -p meta) [36] and used the CD-HIT default parameters (v 4.8.1, -G 1 -c 0.9) [37] to render the genes predicted by prodigal de-redundant. Further, nonredundant (NR) predicted genes were compared using Salmon (v 1.2.1, -validate Mappings -meta) [38] with clean data, and the relative abundance of NR-predicted genes (RPM) was calculated. Finally, the transeq command of the EMBOSS software was used to translate the NR gene into a protein sequence for subsequent alignment and annotation.

### Taxonomic and functional annotation of genes and abundance estimations

Taxonomic classification was performed using Kraken 2 [39] with a confidence interval of 0.2. Bracken [40] was used to predict the relative abundance of taxonomic species in the sample. DIAMONG (v0.9.21.122) [41] with *e*-values ≤ 1e–5 was used to match the amino acid sequences of proteins in the catalog to the UniProt TrEMBL. Proteins that could not be aligned to the database were categorized as unknown proteins. The KEGG annotation results were extracted using KOBAS (v3.0.3) software (-t blastout:tab, -s ko). Using eggNOG-mapper software [42] (based on DIAMOND), the de-redundant protein sequences (corresponding to the nucleic acid sequences of the NR predicted genes) were compared to the EggNOG database to obtain the KEGG, GO, and COG annotation information for the proteins. Comparison parameters: seed ortholog e-value = 0.00001. De-redundant protein sequences were compared with the CAZy database to obtain annotation information for CAZy using DIAMOND [41]. Comparison parameters are as follows: e 0.00001 (*e*-value threshold) –id 80 (identity threshold) –top three (bit score not less than 3% of the highest score). The abundance of redundant predicted genes annotated to the same gene family in the database was summed based on the abundance table of redundant genes and each database annotation information. Furthermore, the redundant predicted genes that failed to match were screened to obtain the relative abundance table of gene families in each database.

### Metagenomic binning and genome quality assessment

We used functionality modules from the previously published metaWRAP pipeline (v1.3.2) to perform genome assembly of the fecal microbiomes from the sequencing data [43]. For the sheep and goat microbiomes, individual sample overlapping clusters was constructed. First, the quality (length) of each metagenome was filtered using the sequencing read option “–min-contig-len 500,” resulting in 2,681,185 contigs with a length of 1.73 × 10^10^ bp and an N50 value of 20,669 bp. The clean reads were then aligned to contigs using Bowtie 2 (v2.3.5.1) [34] with default parameters, and the resulting alignment files were sorted and indexed using SAMtools (v1.9). The sorted BAM files were used to calculate the corresponding contig depths using the jgi_summarize_bam_contig_ depth function in MetaBAT 2 (v.2.12.1) [44].

We used the metaBAT v 2.12.1 (-m 1500 and-unbinned parameters) [44] and MaxBin v2.2.6 [45] (-markerset 40 option) modules in the binning module to assemble the genomes. The Bin_refinement module uses a hybrid approach to generate a consolidated and improved bin set by evaluating two or three sets of bins obtained from different binning approaches. The bin sets were initially hybridized using binning_refiner v1.2 (default settings) to create bin sets AB, BC, AC, and ABC when the three original bin sets, A, B, and C, were present. The “MetaWRAP-Reassemble_bins” module was then used to improve the bin set by extracting and reassembling the readings belonging to each bin. This involved indexing the entire original metagenomic assembly and aligning FastQ reads using BWA v0.7.15 (default parameters). Moreover, even if only one read was aligned, reads mapped back to contigs in the specified bins were kept in separate FastQ files [46]. Two sets of reads were stored in each bin: reads mapped perfectly (strict) and reads mapped with < 3 mismatches (permissive). Each set of reads was then reassembled using SPAdes v3.11 (–careful setting) [47], and short contigs (less than 1000 bp) were removed. The standard workflow of CheckM v1.0.12 (default settings) [48] was used to assess the completion and contamination of each bin version, with completeness criteria between 50 and 90% and contamination ≤ 5%. Genomes meeting the completeness ≥ 90% and contamination ≥ 5% criteria were classified as high-quality genomes, whereas those with completeness between 50 and 90% and contamination ≥ 5% were classified as medium-quality genomes. The coverage of each contig was calculated using the “coverage” command in CheckM. The resulting MAGs were clustered into species-level genome bins (SGBs) using the “dereplicate” program in dRep (v3.2.2) [49] with a threshold of > 90% average nucleotide identity (ANI). SGBs containing at least one reference genome (or metagenome-assembled genome) in the Genome Taxonomy Database (GTDB, https://gtdb.ecogenomic.org/) were considered known SGBs, while those without a reference genome were classified as uSGBs.

### Phylogenetic, taxonomic, and functional analyses of genomes

To construct phylogenetic trees, the “classify” workflow in GTDB-Tk (v.2.1.0; default settings) [50] was used to identify MAGs (5810), bacterial (62,291), and archaeal marker genes (3412). Based on the archaeal marker genes and bacteria above from the GTDB database (reference database version R207) [50], a multiple sequence alignment was built. The resulting FASTA files containing multiple sequence alignments of the submitted genomes were used for maximum likelihood phylogenetic tree inference using IQ-TREE (v.1.6.11) [51]. iTOL (v.5.6.2) was used to visualize phylogenetic tree output [52]. Overall, 5843 MAGs were classified using GTDB-tk [50]. The total branch length of the MAGs was calculated and compared to the total branch length of the entire bacterial tree to estimate the increase in phylogenetic diversity (PD) caused by the inclusion of the MAGs, and the increase in the phylogenetic tree was calculated using “phytools” [24].

The MetaWRAP Quant_bins module was used to estimate the relative abundance of each bin in each sample [43]. Salmon v 1.10 (–libType IU option) [38] was used to index the entire metagenomic assembly and align the reads from each sample to the assembly. A coverage table was generated to estimate the abundance of each contig fragment in each sample. The length-weighted average of contig abundances within the bin was determined to obtain the average abundance of each bin in each sample. The following parameters were used for screening sheep-specific MAGs: goat ratio = 0 and sheep ratio > uniqratio, where the minimum value for uniqratio was set to 0.2. To predict CAZymes [53] from 5810 MAGs, hmmsearch (v3.1) [54] was run against dbCAN HMMs (v8) [55], and an *e*-value cutoff of < 1 × 10^−5^ was used. The PUL of all MAGs was predicted using the PULpy (v.1.0) [56] pipeline.

### Statistical analysis

The pre-description method was used to analyze the rarefaction curve [57]. Vegan in the R package (v3.6.1) was used to calculate the diversity of the gut microbiota, including the number of observed species, Shannon index, and principal coordinate analysis based on the Bray–Curtis distance. PERMANOVA (default options) implemented in the R package “vegan” was used to investigate the effects of host and rearing systems on the microbiota composition. We performed both single- (i.e., consider host) and multifactor (i.e., considering host and rearing system) analyses. Differences in *α*-diversity were compared using the one-tailed Wilcoxon rank-sum test with FDR correction. To compare the gut microbiomes between sheep and goats or between the grazing and drylot groups of goats, we performed pairwise comparisons using a pairwise Wilcoxon test and two-tailed Fisher’s test with FDR correction under the premise of fixed host or feeding environmental factors. The PCoA model utilized PERMANOVA implemented in R package “vegan.” Significance thresholds for comparisons of taxa, KEGG pathways, and MAGs between sheep and goats were set at *P* < 0.001 after Bonferroni correction. The results were represented using boxplots or heat maps created using the ggpubr and pheatmap packages in R (v3.6.1).

## Results

### Construction of sheep and goat gut microbial gene catalogs

We collected 320 fecal samples from 210 sheep and 110 goats, representing 21 breeds from 32 farms (Fig. 1a). Sheep and goats varied in sex and age and were fed different diets under different conditions (Additional file 2: Table S1). We extracted all DNA samples and sequenced the metagenome using the Illumina NovaSeq 6000 platform, yielding 7.8 terabyte (Tb) of high-quality data at an average of 24.5 gigabyte (Gb) per sample. According to the data processing workflow (Additional file 1: Fig. S1), Rarefaction analysis suggested that the number of SMGC and GMGC clusters approached a saturation point (Fig. 1b). We identified 162,518,163 and 82,982,669 NR-predicted genes with average N50 contig lengths of 660 and 678 kb in sheep and goat samples, respectively (Fig. 1b; Additional file 3: Table S2). Furthermore, using this data, we constructed two gut microbiota-predicted gene catalogs for sheep and goats, termed sheep microbial gene catalog (SMGC) and the goat microbial gene catalog (GMGC), respectively. To reduce duplicate functional genes in SMGC and GMGC, these predicted genes were clustered at the protein level using the UniRef model [58] at 100% and 90% amino acid identity to form SMGC100, SMGC90, GMGC100, and GMGC90, respectively. A total of 57.4% and 47.8% known proteins in SMGC90 and GMGC90 was found to be decreased, respectively. Therefore, SMGC100 and GMGC100 were used for subsequent analyses.

### SMGC and GMGC quality and completeness

We further aligned previously published gut metagenomic data generated by Zhang et al. [59] and Zhu et al. [60] for Tibetan goats *(Capra hircus)* and wild blue sheep (*Pseudois nayaur*) to SMGC and GMGC. We found that SMGC and GMGC improved sequencing read mapping, with an average mapping rate of 79.22% and 79.60%, respectively (Fig. 1c). By comparing the gene pairwise overlap of SMGC and GMGC, we determined that over 113,286,370 (69.7%) and 33,750,876 (40.7%) of predicted genes were unique to sheep and goats, respectively, whereas 49,231,793 (30.3% in sheep and 59.3% in goats) of predicted genes are shared between the two species (Fig. 1d). Furthermore, all 210 sheep samples shared a common set of 678 NR-predicted genes (Additional file 4: Table S3), 433 species (Additional file 4: Table S4), 125 carbohydrate-active enzymes (CAZyme) families (Additional file 4: Table S5), and 424 Kyoto Encyclopedia of Genes and Genomes (KEGG) functional pathways (Fig. 1e; Additional file 4: Table S6). Using a cutoff that required a core be shared by 50% of the sheep, the common sets of 7,426,924 NR-predicted genes, 1130 species, and 432 KEGG functional pathways were identified (Fig. 1e). Similarly, we discovered that all 110 goat samples shared 261 NR-predicted genes (Additional file 5: Table S7), 396 species (Additional file 5: Table S8), 114 CAZyme families (Additional file 5: Table S9), and 420 KEGG functional pathways (Fig. 1e; Additional file 5: Table S10). Using a cutoff that required a core be shared by 50% of the goats, the common sets of 3,927,039 NR predicted genes, 1055 species, and 431 KEGG functional pathways were identified (Fig. 1e).

### Reference-based taxonomic composition of sheep and goat microbiome

DIAMONG was used to align the amino acid sequences of the proteins in SMGC and GMGC to UniProt TrEMBL [41]. Total protein clusters formed were 18,144,220 (SMGC100) and 9,353,760 (GMGC100), and we found that only 9,633,327 (5.9%) and 4,937,125 (5.9%) NR-predicted genes can be blasted to known proteins of the UniProt TrEMBL, respectively (Additional file 1: Fig. S2a). For these protein clusters, only 53.1% and 52.8% of protein clusters could be taxonomically classified in sheep and goats, respectively (Additional file 1: Fig. S2b). A total of 52.2% (SMGC) and 52.1% (GMGC) of the protein clusters were assigned to bacteria, 0.89% (SMGC) and 0.67% (GMGC) of the protein clusters were assigned to archaea (Additional file 1: Fig. S2b), and 46.9% (SMGC) and 47.2% (GMGC) of the protein clusters genes were unknown (Additional file 1: Fig. S2b). We used Kraken [61], an ultrafast metagenomic sequence classifier using exact alignments, to analyze the gut taxonomic composition of sheep and goats. At the phylum level, Firmicutes had the most annotated genes (49.3% and 52.8%), followed by Bacteroidetes (38.1% and 36.1%) and Proteobacteria (3.26% and 2.89%) in sheep and goat samples, respectively (Additional file 1: Fig. S2c). *Candidatus_Melainabacteria* and Spirochaetes were the fifth and sixth most abundant phyla in sheep and goats, respectively (Additional file 1: Fig. S2c).

**Fig. 2:**
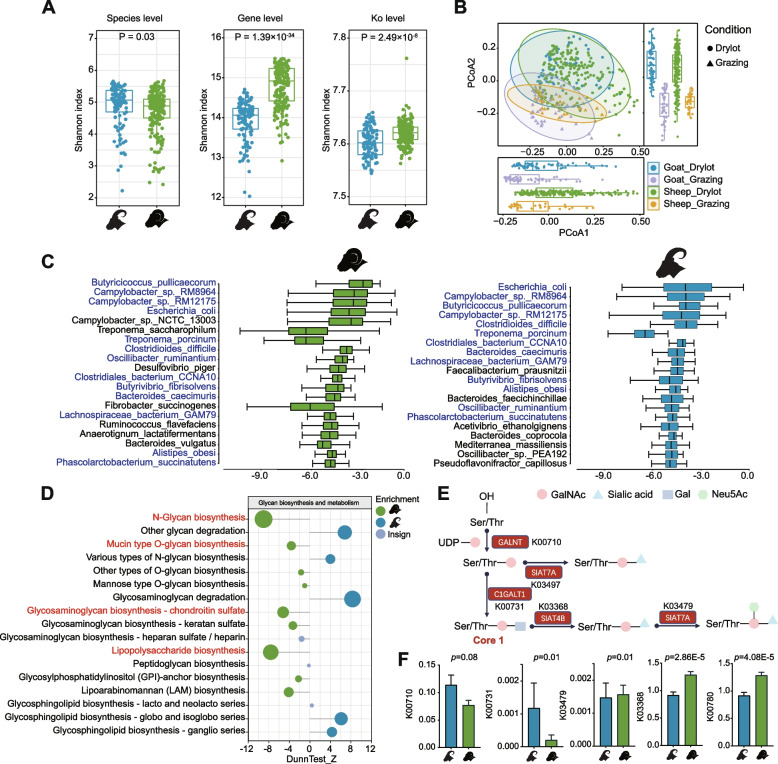
Taxonomic and functional landscape of sheep and goat gut microbiome. **A** Alpha-diversity analysis based on species, genes, and KEGG orthologs. Different colors represent different species, and the Wilcoxon rank-sum test was used for statistical analysis. **B** PCoA plot based on relative species abundances. The colors and shapes of the symbols indicate host species and rearing systems, respectively. Box plots show the Bray–Curtis distances associated with regions and species (Wilcoxon rank-sum test). An analysis of similarity (ANOSIM) was used to assess the dissimilarity of Bray–Curtis. **C** The top 20 bacterial species in sheep and goat guts based on relative abundance. The green color indicates the species in the top 20 lists of sheep, whereas the blue color indicates the species in the top 20 lists of goats. The *x*-axis shows the log10 (relative abundance) values. Blue bacterial names represent the top 20 shared bacteria in sheep and goats. **D** Enrichment differences of related third-level metabolic pathways in the glycan biosynthesis and metabolism pathway based on KEGG analysis in sheep and goats. The green circle represents the metabolic pathway significantly enriched in sheep, the blue circle represents the metabolic pathway significantly enriched in goats, and the gray circle represents no significant difference between the two groups. The Dunn test was used to compare the two groups, with *P* < 0.01 as the significance level. **E** The schematic diagram of mucin-type O-glycan metabolism in the hindgut indicates the critical genes involved in these processes. **F** The pairwise Wilcoxon rank-sum test was used to compare the abundance of important genes involved in mucin-type O-glycan metabolism in sheep and goats

To further assess gut microbial sharing and differential microbial between sheep and goats, a comparison of the gut microbial composition revealed that goats have a higher *α*-diversity than sheep at the species level (*P* = 0.03, Fig. 2a). On the contrary, sheep have a higher *α*-diversity than goats at the NR-predicted genes (*P* < 0.001) and KEGG orthologous group (KO; *P* < 0.001) levels (Fig. 2a). Species-level principal coordinate analysis (PCoA) revealed that while significant differences exist in the gut microbiota composition between sheep and goats under identical rearing conditions (PERMANOVA; drylot sheep *vs*. drylot goats, R2 = 0.041, F = 10.06, *P* < 0.001; grazing sheep *vs*. grazing goats, R2 = 0.162, *F* = 15.15, *P* < 0.001), the same host’s species showed very significant differences in gut microbial composition under different feeding conditions (PERMANOVA; drylot sheep *vs*. grazing sheep, R2 = 0.092, F = 21.24, *P* < 0.001; drylot goats *vs*. grazing goats, R2 = 0.179, F = 23.62, *P* < 0.001; Fig. 2b). We identified 1720 genera in the sheep and goat samples. The 16 most abundant core genera in sheep were also among the top 20 core genera in goats (Additional file 1: Fig. S3b), mainly including *Bacteroides*, *Campylobacter*, *Escherichia*, *Treponema*, *Butyricicoccus*, and *Alistipes*. Further, 1138 species were found in sheep and goat samples, with 138 species found exclusively in goat samples, and 406 species were found only in sheep samples (Additional file 1: Fig. S3a; Additional file 6: Table S11). Importantly, the 13 most abundant core species in goats were also among the top 20 core species in sheep (Fig. 2c), mainly including *Butyricicoccus_pullicaecorum*, *Campylobacter*_sp._RM8964, *Escherichia_coli*, *Treponema_porcinum*, *Campylobacter*_sp._RM12175, and *Clostridioides_difficile* (Fig. 2c). Overall, species enriched in the sheep samples relative to the goat samples included *Corynebacterium freneyi*, *Campylobacter* sp._NCTC_13003, *Bifidobacterium pseudocatenulatum*, *Fibrobacter intestinalis*, and *Bifidobacterium choerinum* [two-tailed Fisher’s test with false discovery rate (FDR) correction, *P* < 0.001; Additional file 1: Fig. S3c–d]. On the other hand, *Pasteurella multocida*, *Cutibacterium avidum*, *Streptococcus equinus*, *Streptomyces gilvigriseus*, and *Corynebacterium kroppenstedtii* are enriched in the goat compared to the sheep samples (two-tailed Fisher’s test with FDR correction, *P* < 0.001; Additional file 1: Fig. S3c–d).

### Functional landscape of sheep and goat gut microbiome

Furthermore, we used the KEGG database to annotate the genes in SMGC and GMGC. We identified 12,388 and 12,247 KOs in sheep and goat samples, respectively. In goats, predicted genes annotated in “carbohydrate metabolism,” “energy metabolism,” “lipid metabolism,” “xenobiotic biodegradation and metabolism,” and “membrane transport” were more prevalent (Additional file 1: Fig. S4a). In contrast, sheep samples contained more genes in “replication, repair, and nucleotide metabolism” relative to the goat samples (Additional file 1: Fig. S4a). The microbiota in sheep were enriched in the glycosyl transferase class based on CAZyme analysis relative to the goat samples. In contrast, microbes in goats were enriched in glycoside hydrolases (GH), carbohydrate-binding modules (CBM), carbohydrate esterases (CE), and polysaccharide lyases (PL) classes relative to the sheep samples (Additional file 1: Fig. S4b). The mechanisms involved in “glycan biosynthesis and metabolism” differ significantly between sheep and goats. The sheep gut was specifically enriched in “N-glycan biosynthesis,” “mucin-type O-glycan biosynthesis,” “glycosaminoglycan biosynthesis-chondroitin sulfate,” and “lipopolysaccharide biosynthesis pathways.” However, “other glycan degradation pathways,” “glycosaminoglycan degradation pathways,” and “various N-glycan biosynthesis pathways” were highly enriched in the goats (Fig. 2d). Mucin is a component of the protective mucous layer of epithelial cells. Thus, we next focused on the mucin-type O-glycan biosynthetic pathway in subsequent analyses. In comparison to sheep, goats had significant upregulation of the *C1GALT1* enzyme gene (K00731), which is involved in the synthesis of the core1 structure of mucin (Fig. 2e–f). However, transferases, such as *SIAT4B* and *SIAT7A* (K03368 and K03479), which further elongate the core1 structure and add neutral and negatively charged sugars (Fig. 2e-f), such as Neu5Ac and sulfated GlcNAc, are abundant in the sheep. In addition, the known mucin-degrading CAZyme families, GH2, GH20 (β-galactosidase), GH89 (α-N-acetylglucosaminidase), and GH29 and GH95 (α-L-fucosidase), were significantly enriched in goat samples relative to the sheep samples, whereas GH33 (sialidase) and GH98 (endo-β1,4-galactosidase) were significantly enriched in the sheep samples relative to the goat samples (Additional file 1: Fig. S4c).

### Host-rearing systems profoundly altered the composition and function of gut microbiota

We have found that the rearing systems significantly affected the gut microbiome composition more than the host species. To reveal these differences in more detail, we compared the distinctions in the gut microbiota composition and functionality of goats under grazing and drylot conditions. At the KO level, the metagenomes of the drylot condition exhibited a higher alpha diversity than the grazing condition (*P* < 0.001; Additional file 1: Fig. S5a). However, there was no significant difference at the species or gene levels (*P* > 0.05; Additional file 1: Fig. S5a). Species- and KO-level PCoA analysis revealed that the grazing samples differ significantly from the drylot samples (PERMANOVA; Species-level: grazing *vs*. drylot, R2 = 0.179, *F* = 23.62, *P* < 0.001; KO level: grazing *vs*. drylot, R2 = 0.256, *F* = 37.27, *P* < 0.001; Additional file 1: Fig. S5b). In goat samples, *Escherichia coli* (*P* < 0.001) and *Alistipes obesi* (*P* < 0.001) are enriched in the grazing samples compared to drylot samples (Additional file 1: Fig. S5c). In contrast, *Butyricicoccus pullicaecorum*, *Clostridioides difficile*, and *Butyrivibrio fibrisolvens* (*P* < 0.001) and members of the *Butyrivibrio* and *Butyricicoccus* genera are enriched in the drylot samples compared to grazing samples (Additional file 1: Fig. S5c). KEGG analysis revealed that “carbohydrate metabolism,” “glycan biosynthesis and metabolism,” “energy metabolism,” and “lipid metabolism” were enriched in grazing samples, whereas “amino acid metabolism,” “membrane transport,” “infectious diseases,” “parasitic diseases,” and “viral diseases” were enriched in drylot samples (Additional file 7: Table S12). We also found that the “*Salmonella* infection pathway” (*P* < 0.001), “*Vibrio cholerae* infection” (*P* = 0.04), and “*Staphylococcus aureus* infection pathway” (*P* < 0.001) were significantly enriched in the drylot samples. Different KOs were involved in “*Salmonella* infection pathway,” including nitric oxide reductase FlRd-NAD reductase (K12265; *norW*; *P* < 0.001), secreted effector protein PipB2 (K15352; *pipB2*; *P* < 0.001), *Salmonella* plasmid virulence protein B (K15366; *spvB*; *P* < 0.001), and nitric oxide dioxygenase (K05916; *hmp*; *P* < 0.001). Different KOs were involved in “*Vibrio cholerae* infection pathway,” including protein transport protein SEC61 subunit gamma and related proteins (K07342; *secE*; *P* < 0.001), vibriolysin (K08604; *nprV*; *P* < 0.001), adenylate cyclase 9 (K08049; *ADCY9*; *P* = 0.001), protein transport protein SEC61 subunit alpha (K10956; *SEC61A*; *P* = 0.001), and zona occludens toxin (K10954; *zot*; *P* = 0.004). Different KOs were involved in “*Staphylococcus aureus* infection pathway,” including phosphatidylglycerol lysyltransferase (K14205; *mprF*; *P* < 0.001), serine-aspartate repeat-containing protein C/D/E (K14194; *sdrC_D_E*; *P* < 0.001), D-alanine-poly ligase subunit 1 (K03367; *dltA*; *P* < 0.001), exfoliative toxin A/B (K11041; *eta*; *P* < 0.001), cationic antimicrobial peptide transport system permease protein (K19080; *vraG*; *P* < 0.001), membrane protein involved in D-alanine export (K03739; *dltB*; *P* = 0.003), iron-regulated surface determinant protein A (K14193; *isdA*; *P* = 0.005), selectin, platelet (K06496; *SELP*; *P* = 0.008), and surface protein G (K14195; *sasG*; *P* = 0.014; Additional file 1: Fig. S5d). In contrast, the “mucin-biosynthesis capacity pathways of peptidoglycan biosynthesis” (*P* < 0.001), “mucin-type O-glycan biosynthesis” (*P* = 0.003), and “various types of N-glycan biosynthesis” (*P* < 0.001) were more abundant in the grazing group (Additional file 1: Fig. S5e).

### Constructing an annotated MAG database of sheep and goats

**Fig. 3:**
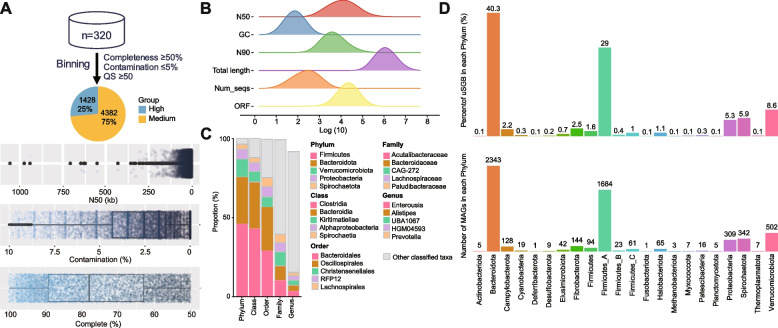
Unknown bacterial genomes identification in sheep and goat gut microbiota. **A** Overall, 5810 MAGs were recovered from sheep and goat hindgut metagenomes. Quality metrics across medium- (*n* = 4,382) and high-quality (*n* = 1428) MAGs. All medium-quality MAGs were ≥ 50% completed and ≤ 5% contaminated, while high-quality MAGs were ≥ 90% completed and ≤ 5% contaminated. **B** The N50, GC, N90, genome sizes, ORF, and the numbers of contigs per genome for the 5810 MAGs, respectively. The *X*-axis shows the log10 values. **C** Taxonomic composition of the 5810 MAGs, ranked from top to bottom by their increasing proportion in the MAGs collection. The legend shows only the five most frequently observed taxa, with the remaining lineages grouped as other classified taxa. **D** The number of all MAGs and the unknown SGB (uSGB) percentage in each phylum. The MAGs without an existing reference genome (which could not be annotated at the species level by GTDB-tk) were defined as uSGBs. The different colors represent phylum

To examine the gut microbiota of sheep and goats comprehensively, we used a metagenomic assembly approach to reconstruct the bacterial and archaeal genomes populating their microbiomes. Using a single-sample assembly strategy optimized to maximize the quality rather than the number of genomes reconstructed from each sample, we reconstructed 9253 MAGs from 110 and 210 metagenome datasets from sheep and goat samples, respectively (Fig. 3a). The CheckM was used to evaluate the quality of MAGs based on the level of MAGs completeness and contamination, and 5810 MAGs of higher than medium quality (≥ 50% completeness and ≤ 5% contamination) were obtained, with 1428 MAGs of high quality (≥ 90% completeness and ≤ 5% contamination). Additionally, we assembled 38 genomes at the strain level (*ANI* ≥ 99%) and 3149 genomes at the species level (*SGBs*, 95% ≥ *ANI* < 99%). Among these, 879 SGBs had high-quality genomes (Fig. 3a; Additional file 8: Table S13). The assembled MAGs ranged in size from 0.33 to 5.34 Mb, with the N50 values ranging from 1.0 kb to 1.1 Mb (Fig. 3b, Additional file 8: Table S13). The 5810 MAGs were then taxonomically classified using the Genome Classification Database Toolkit (GTDB-Tk). At the domain level, all MAGs were classified (5735 for bacteria and 75 for archaea). A vast majority of MAGs (5801; 99.8%) were assigned to the family level, 5597 (96.3%) to the genus level, and only 3149 MAGs to 765 known species (Fig. 3c, Additional file 8: Table S13). The three most frequently assigned families were Acutalibacteraceae (10.74%), Bacteroidaceae (9.16%), and *CAG-272* (8.90%), whereas the top genera were *Enterousia* (3.94%), *Alistipes* (3.72%), and *UBA1067* (3.23%) (Fig. 3c, Additional file 8: Table S13). Importantly, 2661 of the 3149 MAGs representing species without any publicly available genome had ANI values < 95%, indicating the presence of potentially new species defined as unknown SGBs (uSGB), including 40.3% uSGBs belonging to Bacteroidota, 29.0% belonging to Firmicutes_A, and 8.6% belonging to Verrucomicrobiota (Fig. 3d). We obtained 2661 uSGB, indicating potential new strains and species that significantly improved gut microbe coverage in sheep and goats.

**Fig. 4:**
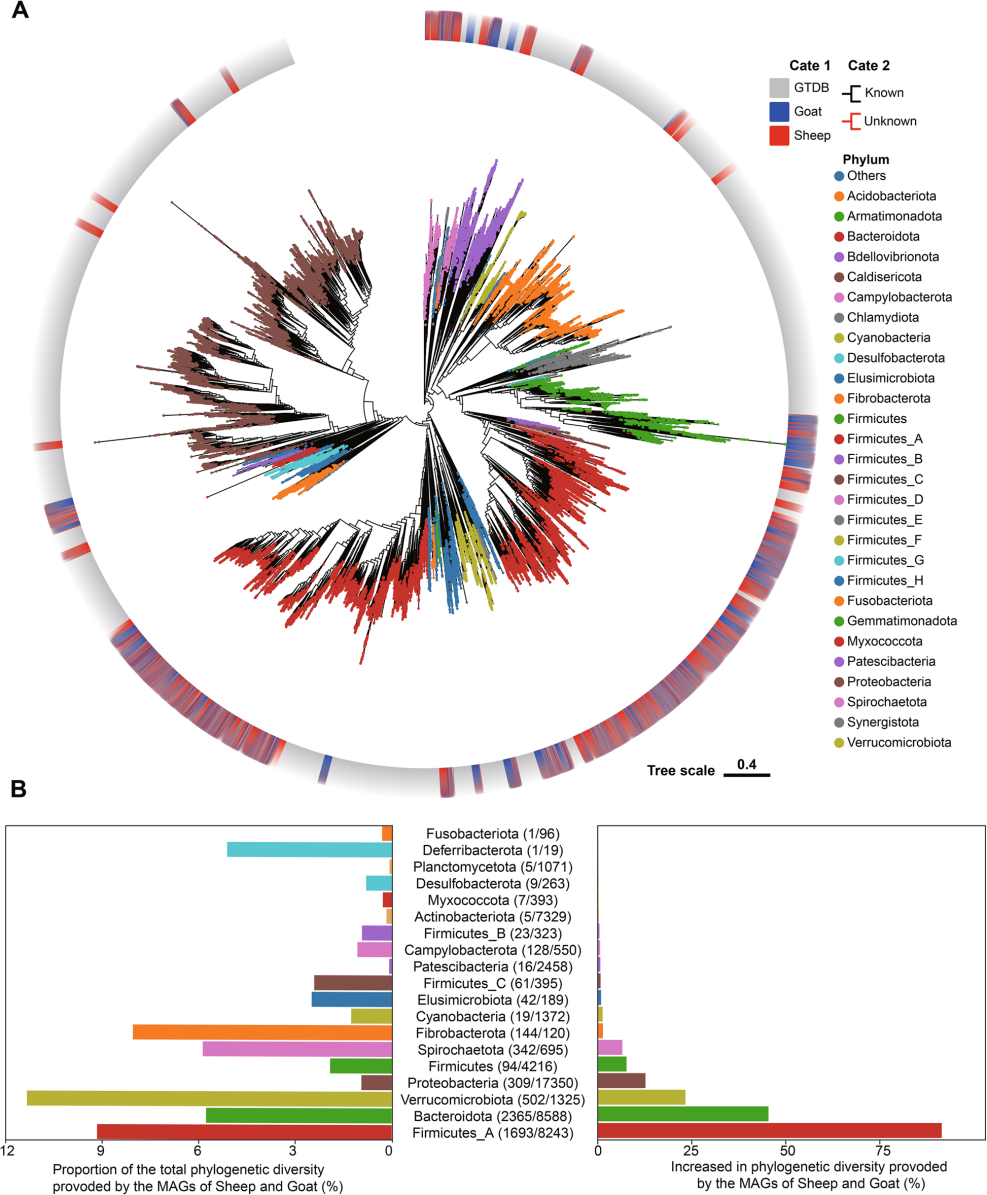
Sheep and goat gut microbial genomes expand the known bacterial phylogenetic diversity. **A** A maximum-likelihood alignment-based phylogenetic tree of the 5735 MAGs assembled in this study and 62,291 bacterial genomes in the GTDB database. Clades are colored based on their phyla. The outer layers contain information on genome sources. Blue represents goat gut genome assembly data, red represents sheep gut genome assembly data, and gray represents GTDB genome data. Dark red indicates clades of unknown SGB. **B** The level of increase in phylogenetic diversity provided by the gut assembly genome set relative to the total diversity per phylum (left) and represented as absolute total branch lengths (right). Brackets depict the number of genomes assigned to each phylum in this study and GTDB (this study/GTDB)

To elucidate whether these MAGs represent new taxa, we compared MAGs to 65,703 prokaryotic genomes (62,291 and 3412 bacterial and archaeal genomes, respectively) from the GTDB database (release date: April 8, 2022). We placed these MAGs in all prokaryotic genomes to better understand the phylogenetic position of the uncultured species in sheep and goats. PhyloPhlAn was used to construct phylogenetic trees (Fig. 4a). Our findings revealed that bacterial MAGs obtained from sheep and goats covered 9.3% of the total phylogenetic diversity in the GDTB database (Fig. 4b), boosting the known bacterial diversity by 2.73% on the basis of total branch length (Fig. 4b). Firmicutes_A and Bacteroidetes exhibited the largest increases. Additionally, the 144 MAGs classified as Fibrobacterota by our assembly were found in only 120 GTDB genome databases (Fig. 4b). Further analysis revealed unknown genomes, a majority of which belonged to Firmicutes, Firmicutes_A, and Bacteroidetes (Fig. 4a). Further analysis of the phylogenetic position of archaea revealed that the MAGs obtained from the assembly in goats were mainly classified as Thermoplasmatota, whereas those obtained from the assembly in sheep were mainly classified as Asgardarchaeota and Methanobacteriota (Additional file 1: Fig. S6a). Importantly, the archaeal MAGs we obtained in sheep and goats increased the known archaeal diversity by 0.21% based on total branch length (Additional file 1: Fig. S6b), which were mainly concentrated in the three clades, including Halobacteriota, Thermoplasmatota, and Methanobacteriota (Additional file 1: Fig. S6b).

### High precision identification of strains with specific colonization in sheep encoding mucin and glycan-metabolizing genes

**Fig. 5:**
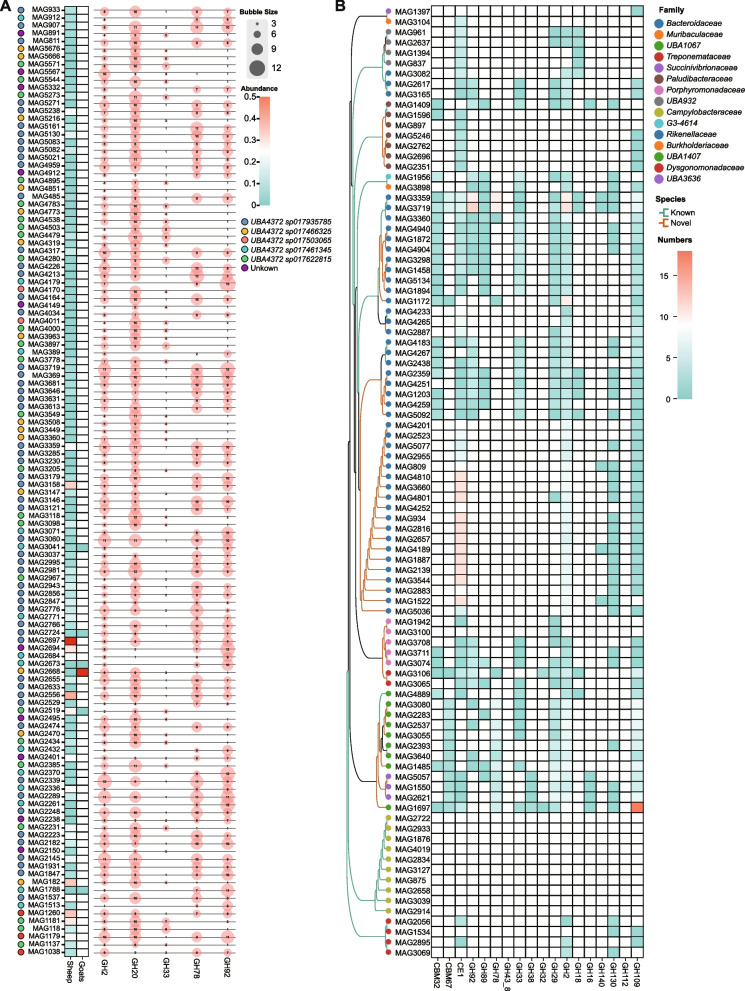
Bacterial and functional characteristics analysis unique to sheep and goat. **A** The number and classification of unique MAGs in sheep and goat guts were analyzed. Circles of different colors represent different bacterial species. The heatmap shows the relative abundance of each MAG in sheep and goat. Blank spaces indicate that the corresponding MAGs were not detected in the gut of that species. The bubble chart represents the number of CAZy enzyme genes encoded by each MAG. **B** Characteristics exploration of 91 MAGs encoding mucin-degrading CAZy genes specifically colonizing in sheep. The classification of the unique 91 MAGs in sheep was analyzed. Circles of different colors represent different bacterial families. The heatmap represents the number of mucin-degrading CAZy enzyme genes encoded by each MAG. Blank spaces indicate that the corresponding enzyme genes were not detected in the genome of these MAGs

In the read-based metagenomic analysis, our preliminary findings suggest the presence of potentially host-specific microbial populations involved in the metabolism of mucin and glycan. To validate this result at the microbial genome level, we next analyzed 5810 medium- to high-quality MAGs and found that most of the 119 MAGs encoded GH2, GH20, and GH78, with a few encoding GH33 and GH92 (Fig. 5a). Similarly, the relative abundance of 122 MAGs classified as *UBA4372* differed between sheep and goats, with MAG2668 being enriched in goats and the remaining 113 MAGs exclusive to sheep but undetectable in goats (Fig. 5a). Most of the 121 enriched MAGs in sheep encoded CAZyme families involved in the degradation of O-glycans and N-glycans, including GH2, GH20, GH78, and GH92, with a few MAGs encoding GH33 (Fig. 5a; Additional file 9: Table S14). We next performed filtering method for MAGs that were detected in > 20% of individuals in either sheep or goats, respectively, and identified 91 MAGs specific to sheep and 1 specific to goats (Additional file 1: Fig. S7; Additional file 10: Table S15). Of them, 91 MAGs covered 14 families. The highest coverage belonged to Bacteroidaceae and Campylobacteraceae, with one specific MAG in goats belonging to the family Muribaculaceae. We then increased the screening threshold to 50%, identifying only four MAGs specifically found in sheep (Additional file 10: Table S15). The four identified microbes were MAG1203 (*HGM04593*), MAG1872 (*Alloprevotella*), MAG3100 (*Porphyromonas*), and MAG4940 (*Alloprevotella*) (Additional file 1: Fig. S8; Additional file 10: Table S15). Three of these microbes belonged to Bacteroidaceae, whereas one belonged to Porphyromonadaceae. Moreover, 37 of the 92 specific MAGs could be annotated with known species data, whereas the remaining 55 represented potential new species (Additional file 10: Table S15). We next analyzed the ability of the 91 MAGs specifically present in the sheep to encode mucin metabolism-related genes (Additional file 1: Fig. S9, Additional file 11: Table S16) and revealed that MAGs categorized as Bacteroidaceae and *UBA1067* encoded multiple mucin metabolism-related genes (Fig. 5b; Additional file 11: Table S16). Eleven MAGs categorized as Bacteroidaceae specifically encoded a high abundance of CE1, MAG1697 encoded a high abundance of GH109, and MAG3359 and MAG3719 encoded a high abundance of GH2, GH92, and GH78, which have identified the primary species and strains involved in the degradation of O-glycans and N-glycans (Fig. 5b, Additional file 11: Table S16).

**Fig. 6:**
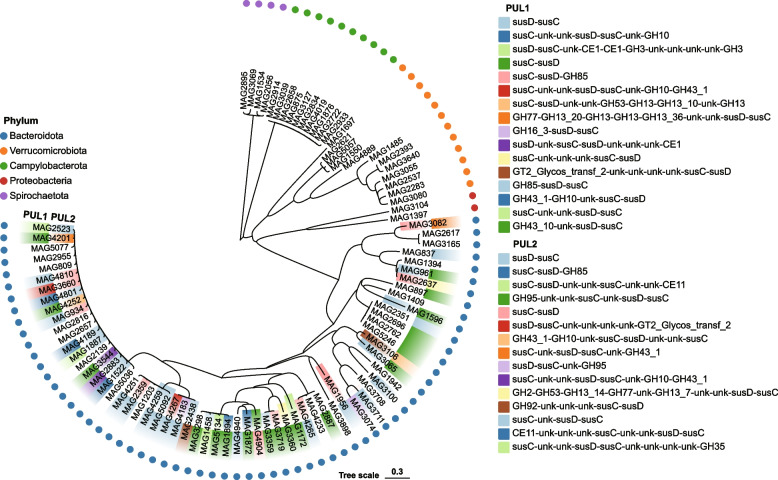
Phylogenetic tree of 91 MAGs specifically colonizing in sheep and its association with polysaccharide utilization loci (PULs). The maximum-likelihood tree of the 91 specifically colonizing in sheep genomes constructed using PhyloPhlAn. Circles of different colors represent different bacterial phyla. Predicted PUL1 and PUL2 in the targeted 91 genomes are presented in the outer and inner layers

To investigate the capacity of 91 bacteria specifically found in sheep for glycan degradation, we predicted the presence of polysaccharide utilization loci (PUL) in the genomes of 91 bacteria specifically found in sheep. Certain Bacteroidetes bacteria, such as MAG4801, MAG4189, and MAG1522, were found to encode the GH10-containing xylanase PUL (Fig. 6; Additional file 12: Table S17). In addition, MAG3106, MAG3082, and MAG3360 encoded the GH13-containing amylase PUL, whereas MAG2523, MAG2883, and MAG1887 encoded the CE1-containing mucoprotease PUL (Fig. 6; Additional file 12: Table S17). In summary, these results further substantiate the potential of host-specific microbial communities in sheep for metabolizing mucin and glycan. More broadly, they demonstrate the potential of exploring functional microbes across diverse animal intestines by constructing microbial assembly genome libraries.

## Discussion

This study successfully obtained comprehensive gene catalogs for the SMGC and GMGC that represent the gut microbiota of sheep and goats, respectively. Employing metagenomic assembly, we generated 5810 medium- and high-quality MAGs. Notably, we identified 91 MAGs specifically colonizing the sheep, prompting a detailed exploration of their functional roles in gut mucin degradation. Our investigation uncovered significant differences in glycan degradation and utilization patterns between the gut microbiota of sheep and goats. This comprehensive analysis enabled us to allocate specific taxa to distinct roles, establishing precise connections between bacteria and their hosts. The importance of our findings is underscored by the novel insights they provide into the symbiotic gut microbiomes of sheep and goats, particularly concerning glycan degradation and its implications for disease susceptibility. Additionally, our study provides valuable data and opens up new avenues for further research into the role of the ruminant gut microbiome in host health and production.

Additionally, we present the largest catalogs of gut microbial genes in sheep and goats to date, encompassing 162,518,163 and 82,982,669 NR-predicted genes, respectively. Less than 6% of the NR-predicted genes could be confidently matched to known proteins. Further comparison with a previously published swine intestinal microbial NR gene set revealed a similar trend, with less than 10% of genes aligning to known proteins [57]. This underscores the likelihood of a proportion of novel, previously unidentified genes within our microbial gene catalogs, which may have been identified through metagenomic predictions but still lack comprehensive functional understanding. Certainly, we acknowledge the potential impact of algorithmic limitations on metagenomic predictions, introducing some level of noise [62], and we also recognize the potential shortfall in coverage within the UniProt TrEMBL database. We plan to curate these unknown genes extensively and establish a dedicated database for unknown genes in the future, facilitating utilization by other researchers. Furthermore, a comparative analysis of our released SMGC with the previously published ruminant gastrointestinal microbial gene catalog (RGMGC) [63], containing 150 million NR genes, reveals that the number of genes in the RGMGC is lower than that in our constructed SMGC. This discrepancy suggests that the SMGC potentially harbors more novel genes. Notably, both the SMGC and GMGC include the most comprehensive array of sheep and goat samples, spanning diverse breeds and habitats, and have been acknowledged for their influence on microbial composition and functional gene content. Validation of the catalogue using data from wild and semi-wild sheep and goat samples showed a match rate of over 79%, confirming the validity of the gene set. We argue that this catalogue is a largely universal and representative tool for elucidating the functions of microbial gene potential in different goat and sheep breeds.

At the microbial taxonomic level, our investigation provides a comprehensive analysis of the common and distinct gut microbial compositions between sheep and goats. Of the top 20 core bacterial species identified in both species, 13 show a consistent presence. This study marks the first comparative study of sheep and goat gut microbiota at the species le vel, prompting us to explore in vitro cultivation techniques to obtain representative microbes. Notably, *Butyricicoccus pullicaecorum* and *Campylobacter* sp. RM8964 are among the top three species in terms of abundance within the sheep and goat gut microbiome. Previous research has highlighted *Butyricicoccus pullicaecorum* as a potential next-generation probiotic, capable of producing high levels of butyrate and exhibiting anti-inflammatory properties [64]. It also has an inherent resistance to low pH and bile acids, which enables its survival and metabolic activity once it reaches the colon [65]. Conversely, bacteria belonging to the genus *Campylobacter* are typically recognized as pathogens [66]. However, despite the high abundance of *Campylobacter* sp. RM8964 in the intestinal tract of healthy sheep and goats, no diarrheal symptoms are observed. Whether the genome of this strain lacks highly virulent genes remains to be determined after isolation and further characterization by in vitro cultivation. These microbial reservoirs hold promise for subsequent functional studies, providing a robust basis for potential industrial applications.

This emphasis originates from the identification of 91 specifically colonizing bacteria in sheep, which are absent in goats. Genomic analysis of these 91 bacteria reveals the presence of enzymes encoding mucin and glycan metabolism, which sparks significant interest. Mucins, heavily glycosylated proteins secreted by intestinal goblet cells, play a pivotal role in shielding the host from direct microbial contact and are regarded as a crucial nutritional source for maintaining stability in the intestinal ecosystem [67]. Under conditions of insufficient dietary carbohydrates, mucins may serve as a nutritional substrate for specific microbes [68]. The judicious utilization of host polysaccharides by the intestinal microbiota proves advantageous, as it may stimulate the excessive production of mucosal polysaccharides, enhancing the protective function of the intestinal barrier [28]. Our findings suggest that, in comparison to goats, the intestinal microbiota of sheep facilitates the elongation of mucin core1 structure into Neu5Ac and sulfated GlcNAc glycosylation structures. Sulfation of mucin glycoproteins contributes to the mucous barrier’s resilience against pathogenic infections and intestinal inflammation [69]. Previous studies have identified specific sulfatase enzymes in *Bacteroides ovatus*, *Bacteroides thetaiotaomicron*, *Bacteroides fragilis*, and *Prevotella RS2* capable of desulfating simple or complex mucin polysaccharides, aiding nutrient acquisition and countering the colonization of other bacteria in the intestine [70, 71]. Our current results strongly suggest notable differences in the mechanisms underlying intestinal inflammation in sheep and goats. To validate these distinctions, further investigations combining glycomics and inflammation models are essential to elucidate the intricate interplay between distinct glycosylation structures and the intestinal microbiota in sheep and goats.

Currently, the dbCAN-PUL database [55] identifies diverse CAZy families implicated in mucin metabolism, encompassing CBM67, CE1 (trehalose 6-O-mycolyltransferase), GH92, GH89 (N-acetylglucosaminidases), GH78, GH43_8, GH33 (sialidases), GH38, GH32, GH29 (fucosidases), GH2 (galactosidases), GH18, GH16, GH140, GH130, GH112, and GH109 [72–74]. After bacterial adhesion to mucins, bacteria expressing glycoside hydrolases include sialidases (GH33), alginate lyases (GH29 and GH95), intramolecular β-galactosidases (GH98), sulfatases (GH20, GH2, and GH42), and core glycoside hydrolases (GH101, GH129, GH84, GH85, and GH89), and microbes encoding these enzymes genes can selectively degrade mucin oligosaccharide chains [29]. In our results, the 91 species specifically colonizing sheep encode GH2, GH20, GH78, and GH92, with a subset of MAGs encoding GH33. This suggests that these microbes may selectively utilize glycan sourced from the sheep host as substrates, enabling specific colonization in the sheep intestine. Hence, it prompts further exploration to unveil differences in mucin secretion and glycosylation structures between sheep and goats. This investigation holds the potential to accelerate our understanding of the precise functions and mechanisms of these specific microbes in intestinal homeostasis and health. Consequently, it provides crucial theoretical data for the targeted utilization of strains in subsequent applications.

We specifically emphasize the significant impact of the rearing environment on the microbial composition and functionality in the goats, surpassing the influence of the host’s species. This conclusion is substantiated by an important body of previous research [75, 76], implicating various factors such as diet and living conditions. Under grazing conditions, goats consume predominantly grass, eliminating the need for supplemental concentrates or silage, resulting in a relatively monotonous diet. This could potentially lead to functional redundancy among microbes, thereby maintaining lower functional diversity. Conversely, in controlled drylot environments, animals are exposed to a wide variety of roughage, concentrates, and silage, requiring gut microbes to have a more diverse functional repertoire to metabolize complex nutritional resources, thereby inducing greater functional diversity. However, it is noteworthy that the controlled drylot environment significantly increases the abundance of pathogenic bacteria (such as *Clostridioides difficile*) in the gut. This results in the significant enrichment of pathways associated with pathogenic bacteria, including *Salmonella* and *Staphylococcus aureus* infection pathway. This may potentially elevate the risk of intestinal diseases in goats. In contrast, under grazing conditions, pathways related to intestinal barrier function, such as the mucin-biosynthesis pathway of peptidoglycan biosynthesis and mucin-type O-glycan biosynthesis, are significantly enriched. This enrichment may reflect a natural protective mechanism against pathogens. Our findings suggest that while controlled drylot environments may enhance the functional diversity and productivity of animal microbiota, they may also lead to an increase in potentially pathogenic bacteria.

Inevitably, notwithstanding its strengths, this study possesses certain limitations. Firstly, the observed differences at the species and KO levels between grazing and drylot samples prompt a more in-depth exploration of potential factors. These disparities primarily encompass influences such as dietary variations, environmental factors, and management practices. Recognizing the inherent complexity of studying complex biological systems, we acknowledge that our study may be subject to certain systematic effects. Potential systematic influences that warrant consideration include sampling bias, where variability in sample collection procedures or the inclusion of animals from different geographic locations, may introduce bias. While providing potential explanations for the differences between grazing and drylot samples, we acknowledge the intricacies of the gut microbiota and the potential impact of various systematic effects on our study results. Further research, encompassing a more comprehensive understanding of dietary, environmental, and management factors, is necessary to validate and extend our findings. In addition, we did not integrate the recently published RGMGC into our analysis, as it became available during the finalization of our study. Thirdly, the microbial communities in other intestinal segments were not examined, constraining the breadth of our findings. Finally, our study excluded the analysis of immature goat or sheep gut contents, despite the availability of a gene set for immature animal guts obtained through amplicon sequencing. Consequently, future investigations should endeavor to assimilate these data and conduct a systematic analysis, thereby constructing a more comprehensive and representative gene set for subsequent applications.

## Conclusions

In conclusion, we present a large-scale annotated bacterial genome database of predominantly unknown species that were extracted from the guts of sheep and goats and identified 91 MAGs that colonized sheep exclusively and encoded enzymes involved in glycan and mucin metabolism. This study will contribute to our profound understanding of the crucial role played by gut microbiota in glycan and mucin metabolism, aiding in the identification of key microorganisms that impact the homeostasis of the intestinal barrier. Overall, our study sheds new insights into the biology and function of sheep and goat gut microbiota, with significant implications for animal husbandry.

### Supplementary Information

**Additional file 1: Figure S1.** Pipeline for the construction of sheep microbial gene catalog (SMGC), goat microbial gene catalog (GMGC) and metagenome-assembled genomes (MAGs). There are two main parts in the pipeline: the construction of the gene catalog and metagenome assembled genomes. We have added the software used and the relevant parameters to the key processes. **Figure S2.** Annotation of genes in the SMGC and GMGC. (A) The number and percentage of the known and unknown proteins in the SMGC and GMGC. A protein was defined as the known protein if its protein sequence could be aligned in Uniprot TrEMBL database. (B) The percentage of genes that could be classified to each taxonomic level in the SMGC and GMGC. (C) The percentage of genes classified different phyla of bacteria. **Figure S3.** Taxonomic landscape of sheep and goat gut microbiome. (A) A total of 1,138 species were found to be present in sheep and goat samples, and 138 species were found only in goat samples, 406 species were found only in sheep samples. (B) The top 20 bacterial genera in relative abundances in sheep and goat, respectively. The green color indicates the genera in the top 20 lists of sheep, and the blue color indicates the genera in the top 20 lists of goats. The log10 (relative abundance) values are shown on the x-axis. Blue bacterial names represent the top 20 shared bacteria in sheep and goat. (C) Differential microbial genus was selected using LEfSe analysis of sheep and goat hindgut. When all samples were treated as independent, the Linear Discriminant Analysis score showed significant enrichment for taxa (*P* < 0.05 and |LDA|> 4). (D) Information on species screened for significant differences in the gut of sheep and goats based on ANCOM analysis, with blue indicating species significantly enriched in the goat, and green representing species significantly enriched in the sheep. **Figure S4.** Functional landscape of sheep and goat gut microbiome. (A) Analysis of differences in gut microbial metabolic function in sheep and goats based on the KEGG pathway. Statistical analysis was performed using Kruskal-Wallis with Bofferoni correction for false discovery rate. (B) Analysis of differences in microbial gut carbohydrase activity in sheep and goats based on the CAZyme database. Statistical analysis was performed using Kruskal-Wallis and corrected for false discovery rate with Bofferoni. (C) Enrichment differences of related CAZyme in sheep and goat. The green circle represents the metabolic pathway significantly enriched in sheep, the blue circle represents the metabolic pathway significantly enriched in goats, and the gray circle represents no significant difference between the two groups. The Dunn Test was used to analyze the differences between the groups, with *P*<0.001 as the significance level. **Figure S5.** Gut microbial composition and function are associated with host rearing systems. (A) Alpha diversity analysis based on species, genes and KEGG orthologs. Different colors represent different rearing systems in goat samples, and the Wilcoxon rank-sum test was used for statistical analysis. (B) PCoA plot based on the relative abundances of rearing systems. The colors and shapes of the symbols indicate rearing systems. Bray-Curtis distances associated with regions and species are shown as box plots (Wilcoxon rank-sum test). The dissimilarity of Bray-Curtis was evaluated by analysis of similarity (ANOSIM). (C) The abundance difference bacterial species in the grazing and drylot systems. (D) The pathway abundance difference of salmonella infection, vibrio cholerae infection, and staphylococcus aureus infection and involved in Kos in the grazing and drylot systems. The Wilcoxon rank-sum test was used for statistical analysis. (D) The pathway abundance difference of peptidoglycan biosynthesis, mucin type O-glycan biosynthesis and various types of N-glycan biosynthesis and involved in KOs in the grazing and drylot systems. The Wilcoxon rank-sum test was used for statistical analysis. **Figure S6.** Gut microbial genomes from sheep and goats expand the known archaea phylogenetic diversity. (A) A maximum-likelihood alignment–based phylogenetic tree of the 75 MAGs assembled in this study and 3,412 archaea genomes in GTDB database. Clades are colored according to phyla. Genome source information is presented in the outer layers. Blue color represents genome assembly data from sheep hindgut, red color represents genome assembly data from goat hindgut, and gray color represents genome data from the GTDB database. Clades of unknown SGB are colored dark red. (B) Level of increase in phylogenetic diversity provided by hindgut assembly genome set in this study, relative to the complete diversity per phylum (left) and represented as absolute total branch lengths (right). The number in this and GTDB genomes assigned to each phylum is depicted in brackets (this study /GTDB). **Figure S7.** Analysis of UBA1067 and functional characteristics unique to the sheep and goats. The number and classification of unique MAGs in the gut of sheep and goats were analyzed. Circles of different colors represent different bacteria species. The heatmap shows the relative abundance of each MAG in the gut of sheep and goats. Blank spaces indicate that the corresponding MAGs were not detected in the gut of that species. The bubble chart represents the number of CAZy enzyme genes encoded by each MAG. **Figure S8.** High precision screening of exclusively colonized strains in the gut of sheep and goats based on the MAG level. Identified 91 MAGs specific to the gut of sheep and one MAG specific to the hindgut of goats. Screening was performed for MAGs that were detected in more than 20% of individuals in either sheep or goats, respectively. Different color MAGs names represent different family levels. MAGs marked with an asterisk represent currently unknown genomes. **Figure S9.** Characteristics exploration of 91 MAGs encoding CAZy genes specifically colonizing in sheep. The classification of the unique 91 MAGs in the gut of sheep was analyzed. Maximum-likelihood tree of the 91 specifically colonizing in sheep genomes constructed using PhyloPhlAn. The heatmap represents the number of mucin-degrading CAZy enzyme genes encoded by each MAG. Blank spaces indicate that the corresponding enzyme genes were not detected in the genome of these MAGs.

**Additional file 2: Table S1.** Background information on the sheep and goat samples.

**Additional file 3: Table S2.** General features of the gene catalogs.

**Additional file 4: Table S3.** The common set of 678 NR genes shared by 100% of sheep. **Table S4.** The common set of 433 species shared by 100% of sheep. **Table S5.** The common set of 125 CAZy family shared by 100% of sheep. **Table S6.** The common set of 424 related KEGG pathway functions shared by 100% of sheep.

**Additional file 5: Table S7.** The common set of 261 NR genes shared by 100% of the goats. **Table S8.** The common set of 396 species shared by 100% of the goats. **Table S9.** The common set of 114 CAZy family shared by 100% of the goats. **Table S10.** The common set of 420 KEGG pathway functions shared by 100% of the goats.

**Additional file 6: Table S11.** Special species were found only in goat and sheep samples.

**Additional file 7: Table S12.** Analysis of KEGG at level 2 of grazing and drylot group in goat samples.

**Additional file 8: Table S13.** The relative abundance of 5810 medium- and high-quality MAGs in sheep and goat gut.

**Additional file 9: Table S14.** The CAZyme-predicted proteins of 5810 medium- and high-quality MAGs in sheep and goat gut.

**Additional file 10: Table S15.** Identified 91 MAGs specific to sheep and one specific to goats.

**Additional file 11: Table S16.** The CAZyme-predicted proteins of 92 MAGs specifically colonizing in sheep and goats.

**Additional file 12: Table S17.** The predicted polysaccharide utilization locus (PULs) of 92 MAGs specifically colonizing in sheep and goats.

## Data Availability

Raw sequence reads for all samples are available under the NCBI project: PRJNA972320 (https://dataview.ncbi.nlm.nih.gov/object/PRJNA972320?reviewer=cbpvcbh5i6pfuqc3cfmv2ck30j). The protein and ORF sequences of all MAGs have been deposited in Figshare (https://figshare.com/s/fe5fb3dd964a15844505). The databases used in this study include GTDB database release 207. The workflow and scripts used to generate the gene catalogs, genomic analysis, and functional annotations are available at https://github.com/bladrome/meta320_binning.

## Abbreviations

MAG: Metagenome-assembled genome
CTAB: Cetyltrimethylammonium bromide
SGBs: Species-level genome bins
ANI: Average nucleotide identity
GTDB: Genome Taxonomy Database
SMGC: Sheep microbial gene catalog
GMGC: Goat microbial gene catalog
NR: Non-redundant
CAZyme: Carbohydrate-active enzymes
GH: Glycoside hydrolases
CBM: Carbohydrate-binding modules
CE: Carbohydrate esterases
PL: Polysaccharide lyases
uSGB: Unknown species-level genome bins
PUL: Polysaccharide utilization loci
RGMGC: Ruminant gastrointestinal microbial gene catalog
