# Supplementary material for: Compendium of 5810 genomes of sheep and goat gut microbiomes provides new insights into the glycan and mucin utilization
Source: Microbiome. 2024 Jun 6;12:104. doi: 10.1186/s40168-024-01806-z (PMC11155115; doi:10.1186/s40168-024-01806-z)
Supplement: Supplementary file 2 — Additional file 1: Figure S1. Pipeline for the construction of sheep microbial gene catalog (SMGC), goat microbial gene catalog (GMGC) and metagenome-assembled genomes (MAGs). There are two main parts in the pipeline: the construction of the gene catalog and metagenome assembled genomes. We have added the software used and the relevant parameters to the key processes. Figure S2. Annotation of genes in the SMGC and GMGC. (A) The number and percentage of the known and unknown proteins in the SMGC and GMGC. A protein was defined as the known protein if its protein sequence could be aligned in Uniprot TrEMBL database. (B) The percentage of genes that could be classified to each taxonomic level in the SMGC and GMGC. (C) The percentage of genes classified different phyla of bacteria. Figure S3. Taxonomic landscape of sheep and goat gut microbiome. (A) A total of 1,138 species were found to be present in sheep and goat samples, and 138 species were found only in goat samples, 406 species were found only in sheep samples. (B) The top 20 bacterial genera in relative abundances in sheep and goat, respectively. The green color indicates the genera in the top 20 lists of sheep, and the blue color indicates the genera in the top 20 lists of goats. The log10 (relative abundance) values are shown on the x-axis. Blue bacterial names represent the top 20 shared bacteria in sheep and goat. (C) Differential microbial genus was selected using LEfSe analysis of sheep and goat hindgut. When all samples were treated as independent, the Linear Discriminant Analysis score showed significant enrichment for taxa (P < 0.05 and |LDA|> 4). (D) Information on species screened for significant differences in the gut of sheep and goats based on ANCOM analysis, with blue indicating species significantly enriched in the goat, and green representing species significantly enriched in the sheep. Figure S4. Functional landscape of sheep and goat gut microbiome. (A) Analysis of differences in gut microbial [file 40168_2024_1806_MOESM1_ESM.docx]

**Supplementary information**

**Compendium of 5,810 genomes of sheep and goat gut microbiome provides new insights into the utilization of** **glycan and mucin**

Ke Zhang^1†^, Chong He^2†^, Lei Wang^3†^, Langda Suo^4,5†^, Mengmeng Guo^6†^, Jiazhong Guo^7^, Ting Zhang^1^, Yangbin Xu^1^, Yu Lei^1^, Gongwei Liu^1^, Quan Qian^1^, Yunrui Mao^1^, Peter Kalds^1^, Yujiang Wu^4,5^, Awang Cuoji^4,5^, Yuxin Yang^1^, Daniel Brugger^8^, Shangquan Gan^9^, Meili Wang^2^, Xiaolong Wang^1,10,11^*, Fangqing Zhao^12^*, Yulin Chen^1,10,11^*

*^1^**International Joint Agriculture Research Center for Animal Bio-breeding, Ministry of Agriculture and Rural Affairs/Key Laboratory of Animal Genetics, Breeding and Reproduction of Shaanxi Province,* *College of Animal Science and Technology, Northwest A&F University, Yangling, 712100, China**;*

*^2^College of Information Engineering,* *Northwest A&F University, Yangling, 712100, China;*

*^3^Plateau Livestock Genetic Resources Protection and Innovative Utilization Key Laboratory of Qinghai Province, Key Laboratory of Animal Genetics and Breeding on Tibetan Plateau, Ministry of Agriculture and Rural Affairs, Qinghai Academy of Animal and Veterinary Medicine, Qinghai University, Xining, 810016, China;*

*^4^Institute of Animal Sciences, Tibet Academy of Agricultural and Animal Husbandry Sciences, Lhasa, 850009, China**;*

^5^*Key Laboratory of Animal Genetics and Breeding on Tibetan Plateau, Ministry of Agriculture and Rural Affairs, Lhasa, 850009, China;*

*^6^* *College of Animal Engineering, Yangling Vocational and Technical College, Yangling, 712100, China*

*^7^College of Animal Science and Technology, Sichuan Agricultural University, Chengdu, China;*

*^8^Institute of Animal Nutrition and Dietetics, Vetsuisse-Faculty, University of Zurich, Zurich, Switzerland;*

*^9^College of Coastal Agricultural Sciences, Guangdong Ocean University, Zhanjiang 524088, China;*

^10^*Key Laboratory of Livestock Biology, Northwest A&F University, Yangling, 712100, China;*

^11^*School of Future Technology on Bio-breeding, Northwest A&F University, Yangling, 712100, China;*

*^12^Computational Genomics Lab,* *Beijing Institutes of Life Science, Chinese Academy of Sciences, Beijing, China.*

^†^These authors contributed equally to this work.

*Correspondence: [xiaolongwang@nwafu.edu.cn](mailto:xiaolongwang@nwafu.edu.cn); [zhfq@biols.ac.cn](mailto:zhfq@biols.ac.cn); [chenyulin@nwafu.edu.cn](mailto:chenyulin@nwafu.edu.cn).

**Supplementary Figures**


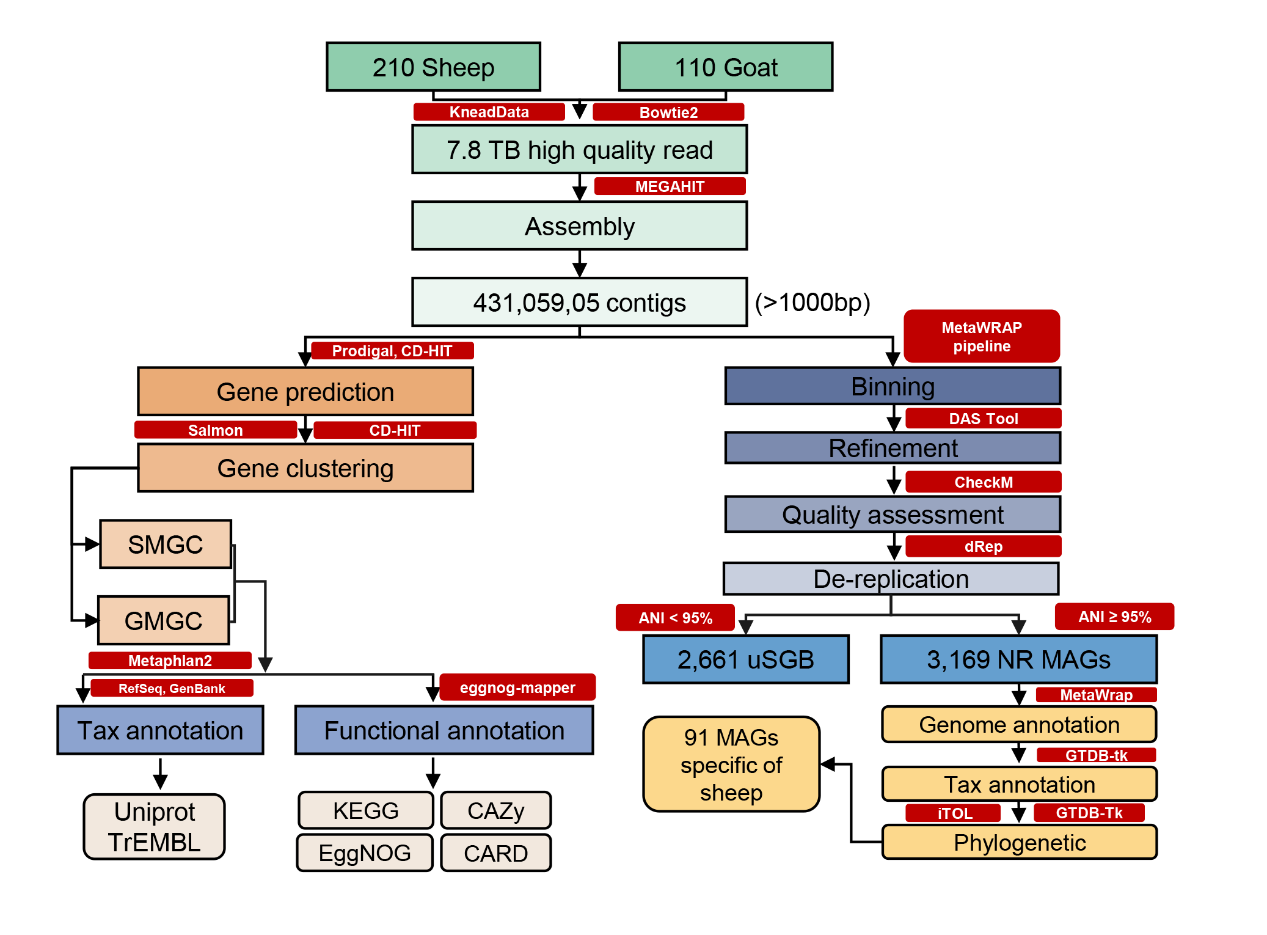


**Figure S1. Pipeline for the construction of sheep microbial gene catalog (SMGC), goat microbial gene catalog (GMGC) and metagenome-assembled genomes (MAGs).** There are two main parts in the pipeline: the construction of the gene catalog and metagenome assembled genomes. We have added the software used and the relevant parameters to the key processes.

**
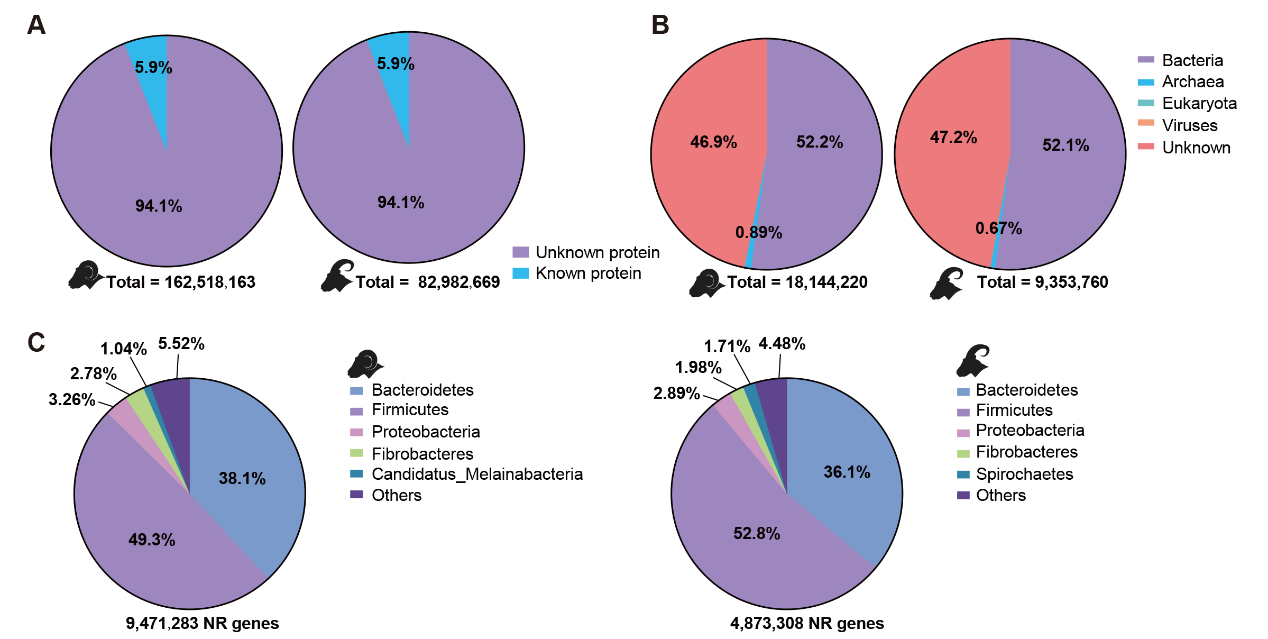
**

**Figure S2. Annotation of genes in the SMGC and GMGC. (A)** The number and percentage of the known and unknown proteins in the SMGC and GMGC. A protein was defined as the known protein if its protein sequence could be aligned in Uniprot TrEMBL database. **(B)** The percentage of genes that could be classified to each taxonomic level in the SMGC and GMGC. **(C)** The percentage of genes classified different phyla of bacteria.

**
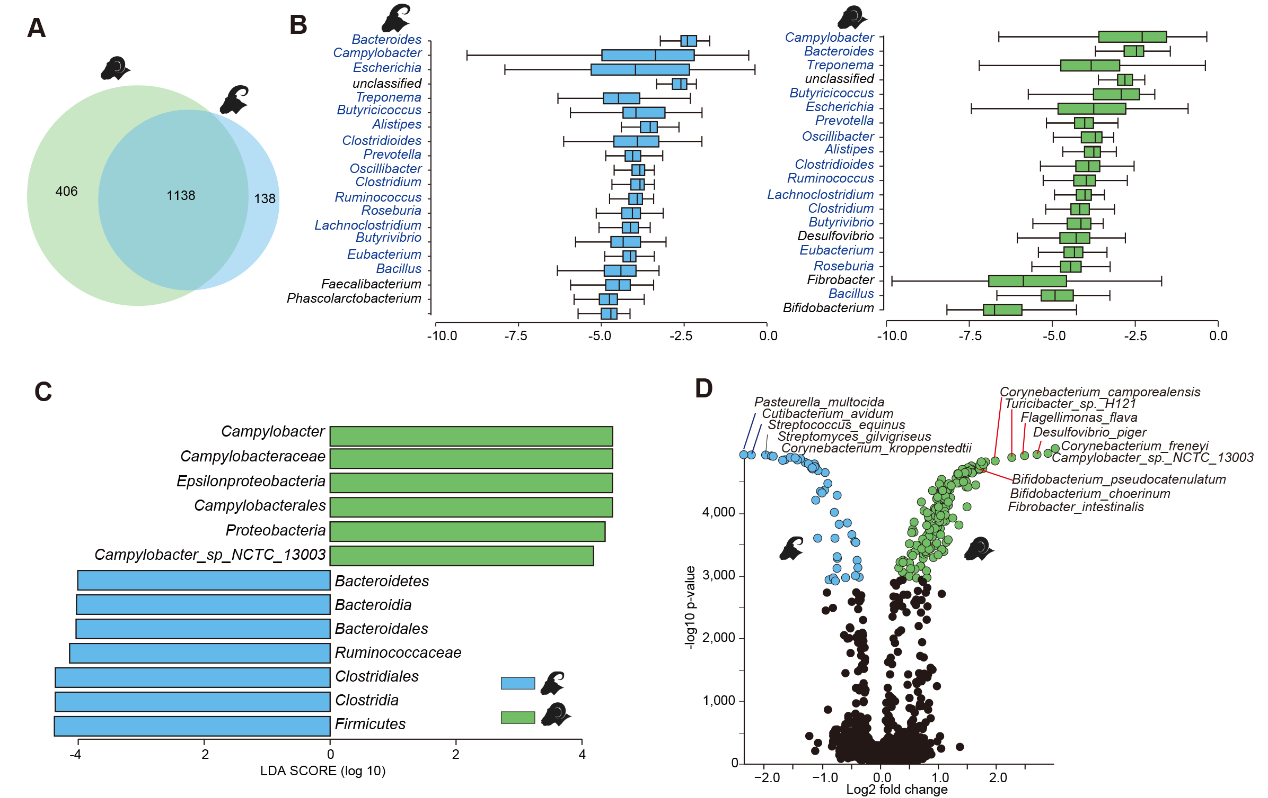
**

**Figure S3. Taxonomic landscape of sheep and goat gut microbiome. (A)** A total of 1,138 species were found to be present in sheep and goat samples, and 138 species were found only in goat samples, 406 species were found only in sheep samples. **(B)** The top 20 bacterial genera in relative abundances in sheep and goat, respectively. The green color indicates the genera in the top 20 lists of sheep, and the blue color indicates the genera in the top 20 lists of goats. The log10 (relative abundance) values are shown on the x-axis. Blue bacterial names represent the top 20 shared bacteria in sheep and goat. **(C)** Differential microbial genus was selected using LEfSe analysis of sheep and goat hindgut. When all samples were treated as independent, the Linear Discriminant Analysis score showed significant enrichment for taxa (*P* < 0.05 and |LDA|> 4). **(D)** Information on species screened for significant differences in the gut of sheep and goats based on ANCOM analysis, with blue indicating species significantly enriched in the goat, and green representing species significantly enriched in the sheep.

**
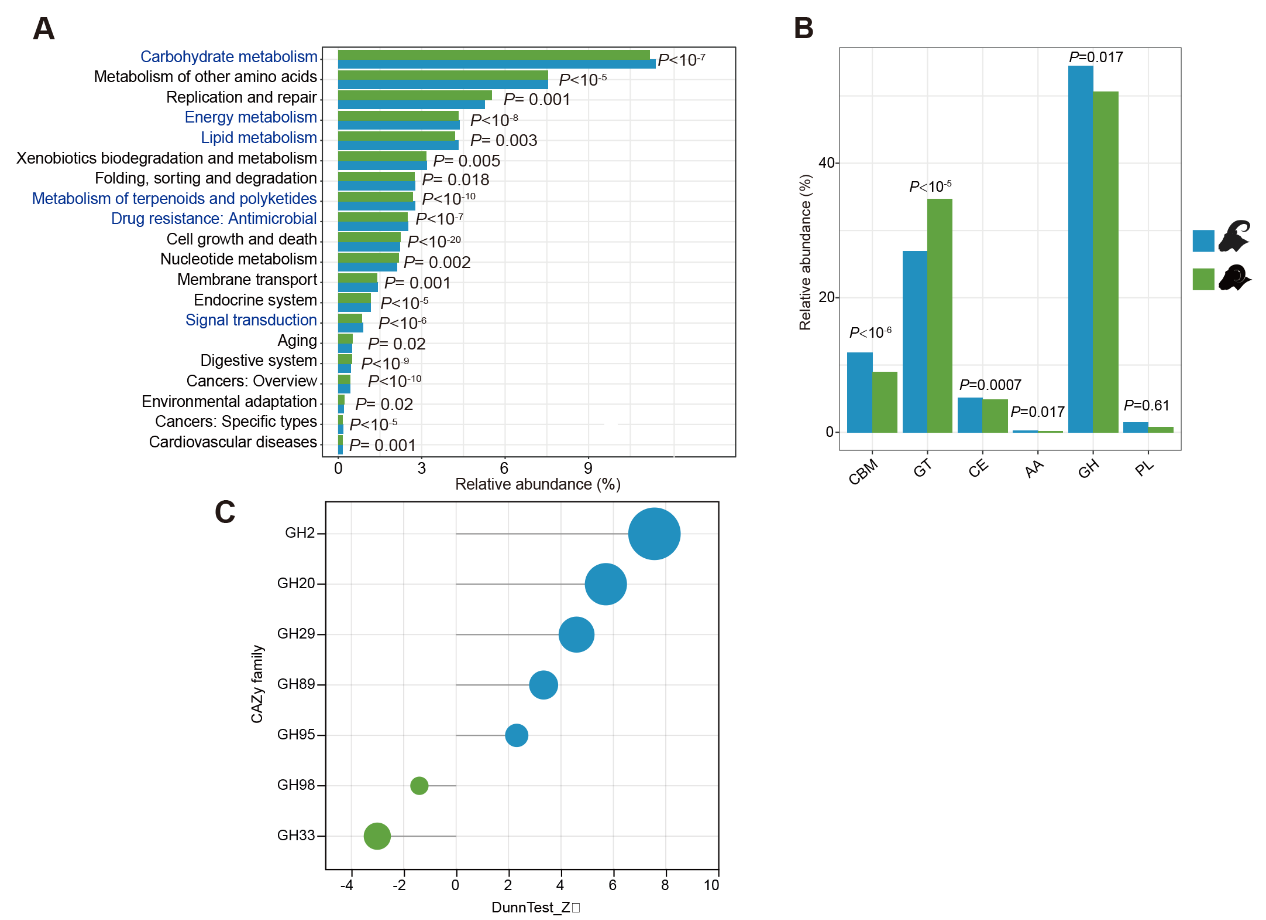
**

**Figure S4. Functional landscape of sheep and goat gut microbiome.** **(A)** Analysis of differences in gut microbial metabolic function in sheep and goats based on the KEGG pathway. Statistical analysis was performed using Kruskal-Wallis with Bofferoni correction for false discovery rate. (B) Analysis of differences in microbial gut carbohydrase activity in sheep and goats based on the CAZyme database. Statistical analysis was performed using Kruskal-Wallis and corrected for false discovery rate with Bofferoni. (C) Enrichment differences of related CAZyme in sheep and goat. The green circle represents the metabolic pathway significantly enriched in sheep, the blue circle represents the metabolic pathway significantly enriched in goats, and the gray circle represents no significant difference between the two groups. The Dunn Test was used to analyze the differences between the groups, with *P*<0.001 as the significance level.

**
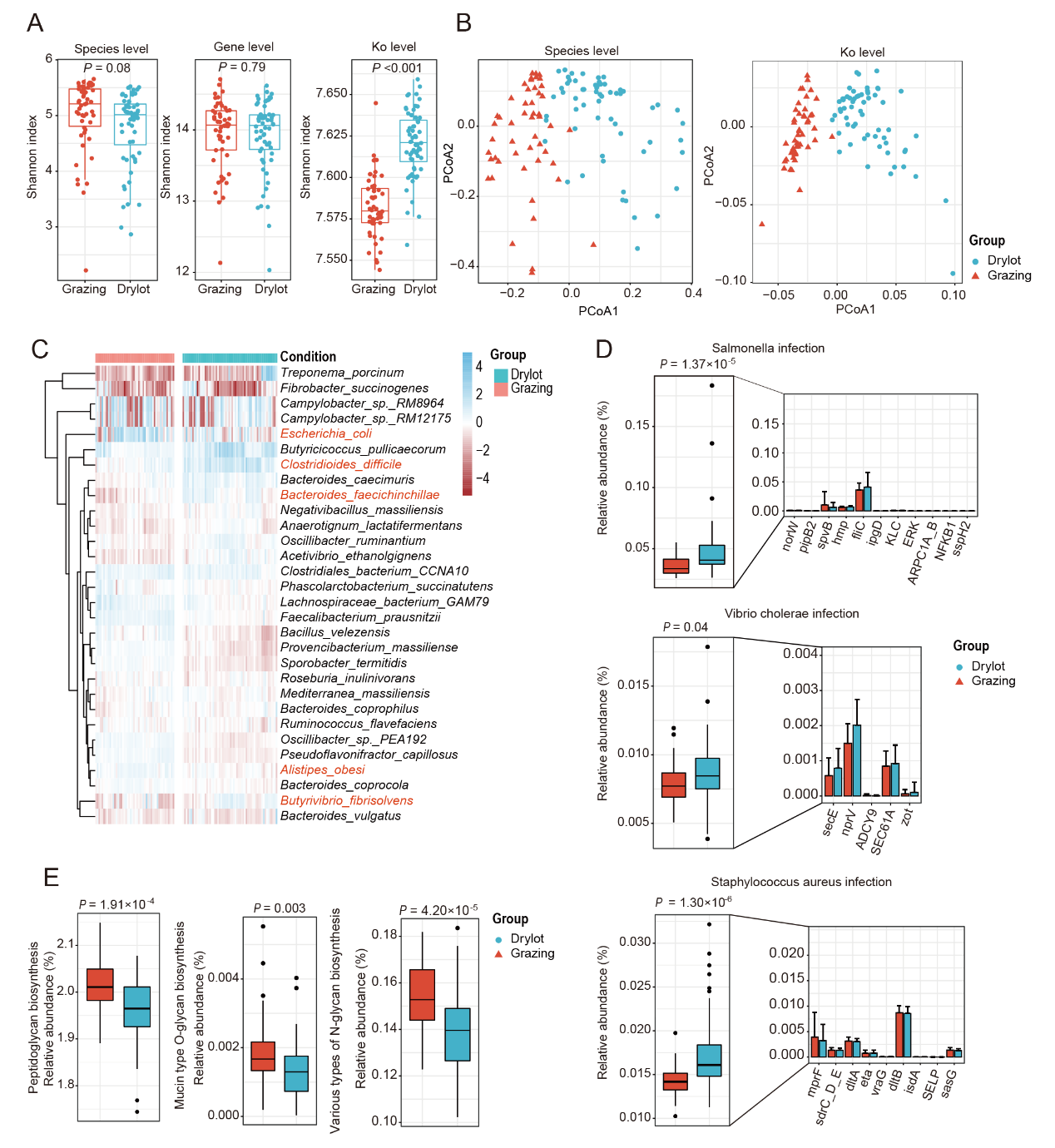
**

**Figure S5. Gut microbial composition and function are associated with host rearing systems. (A)** Alpha diversity analysis based on species, genes and KEGG orthologs. Different colors represent different rearing systems in goat samples, and the Wilcoxon rank-sum test was used for statistical analysis. **(B)** PCoA plot based on the relative abundances of rearing systems. The colors and shapes of the symbols indicate rearing systems. Bray-Curtis distances associated with regions and species are shown as box plots (Wilcoxon rank-sum test). The dissimilarity of Bray-Curtis was evaluated by analysis of similarity (ANOSIM). **(C)** The abundance difference bacterial species in the grazing and drylot systems. **(D)** The pathway abundance difference of salmonella infection, vibrio cholerae infection, and staphylococcus aureus infection and involved in Kos in the grazing and drylot systems. The Wilcoxon rank-sum test was used for statistical analysis. **(D)** The pathway abundance difference of peptidoglycan biosynthesis, mucin type O-glycan biosynthesis and various types of N-glycan biosynthesis and involved in KOs in the grazing and drylot systems. The Wilcoxon rank-sum test was used for statistical analysis.

**
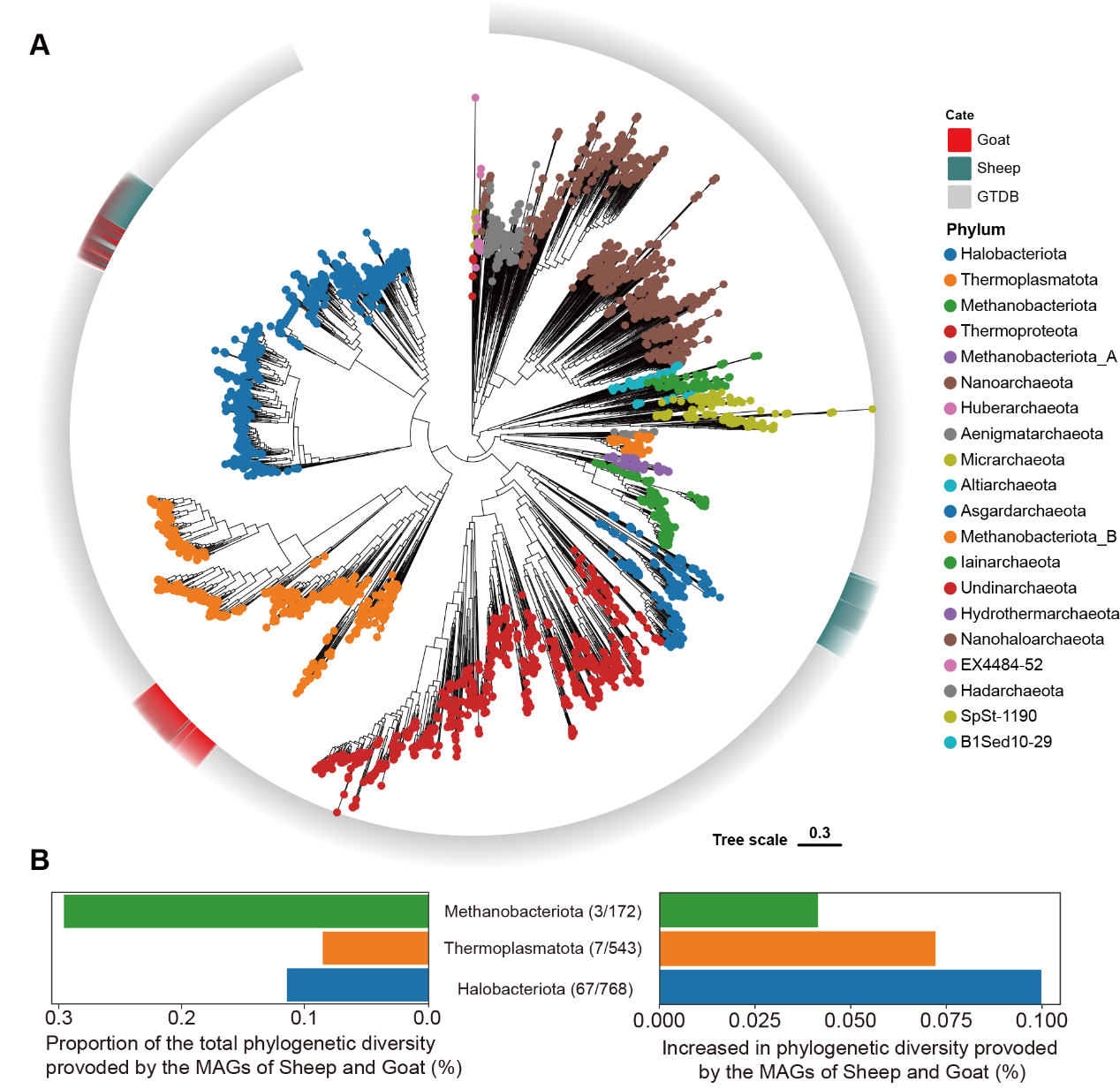
**

**Figure S6. Gut microbial genomes from sheep and goats expand the known archaea phylogenetic diversity.**

(A) A maximum-likelihood alignment–based phylogenetic tree of the 75 MAGs assembled in this study and 3,412 archaea genomes in GTDB database. Clades are colored according to phyla. Genome source information is presented in the outer layers. Blue color represents genome assembly data from sheep hindgut, red color represents genome assembly data from goat hindgut, and gray color represents genome data from the GTDB database. Clades of unknown SGB are colored dark red. (B) Level of increase in phylogenetic diversity provided by hindgut assembly genome set in this study, relative to the complete diversity per phylum (left) and represented as absolute total branch lengths (right). The number in this and GTDB genomes assigned to each phylum is depicted in brackets (this study /GTDB).

**
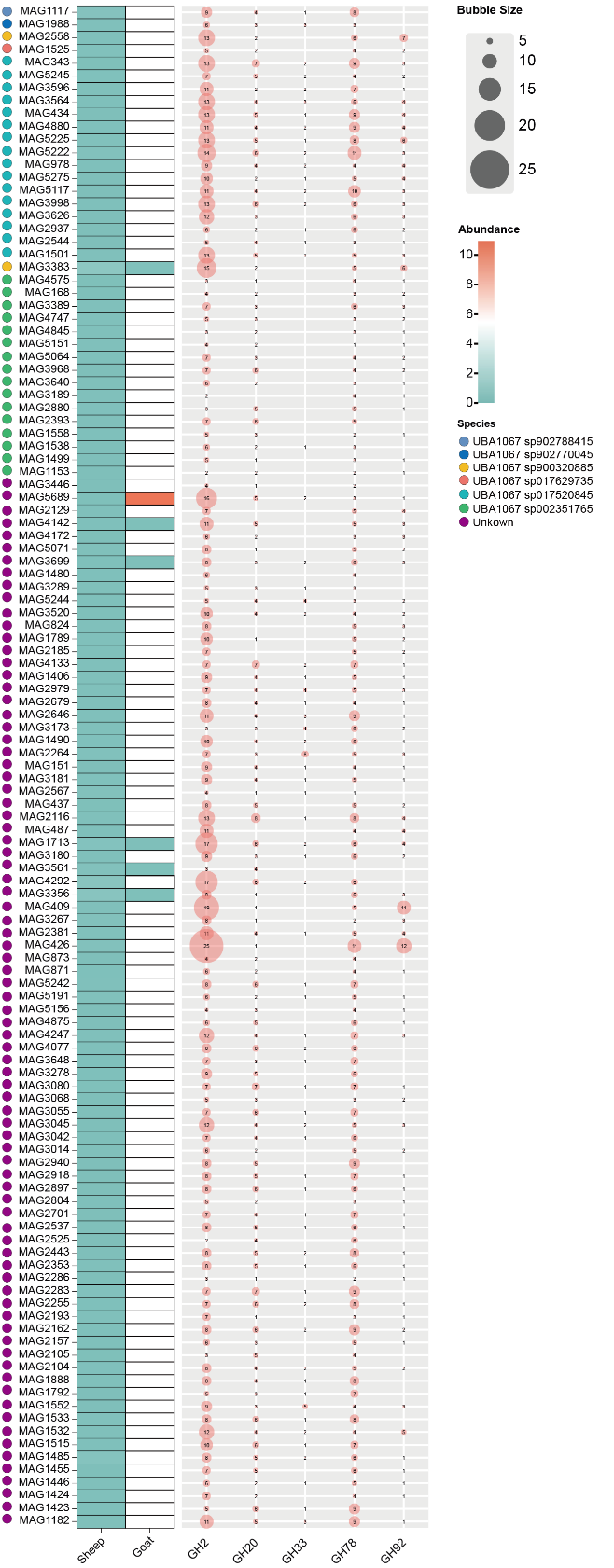
Figure S7. Analysis of UBA1067 and functional characteristics unique to the sheep and goats.** The number and classification of unique MAGs in the gut of sheep and goats were analyzed. Circles of different colors represent different bacteria species. The heatmap shows the relative abundance of each MAG in the gut of sheep and goats. Blank spaces indicate that the corresponding MAGs were not detected in the gut of that species. The bubble chart represents the number of CAZy enzyme genes encoded by each MAG.

**
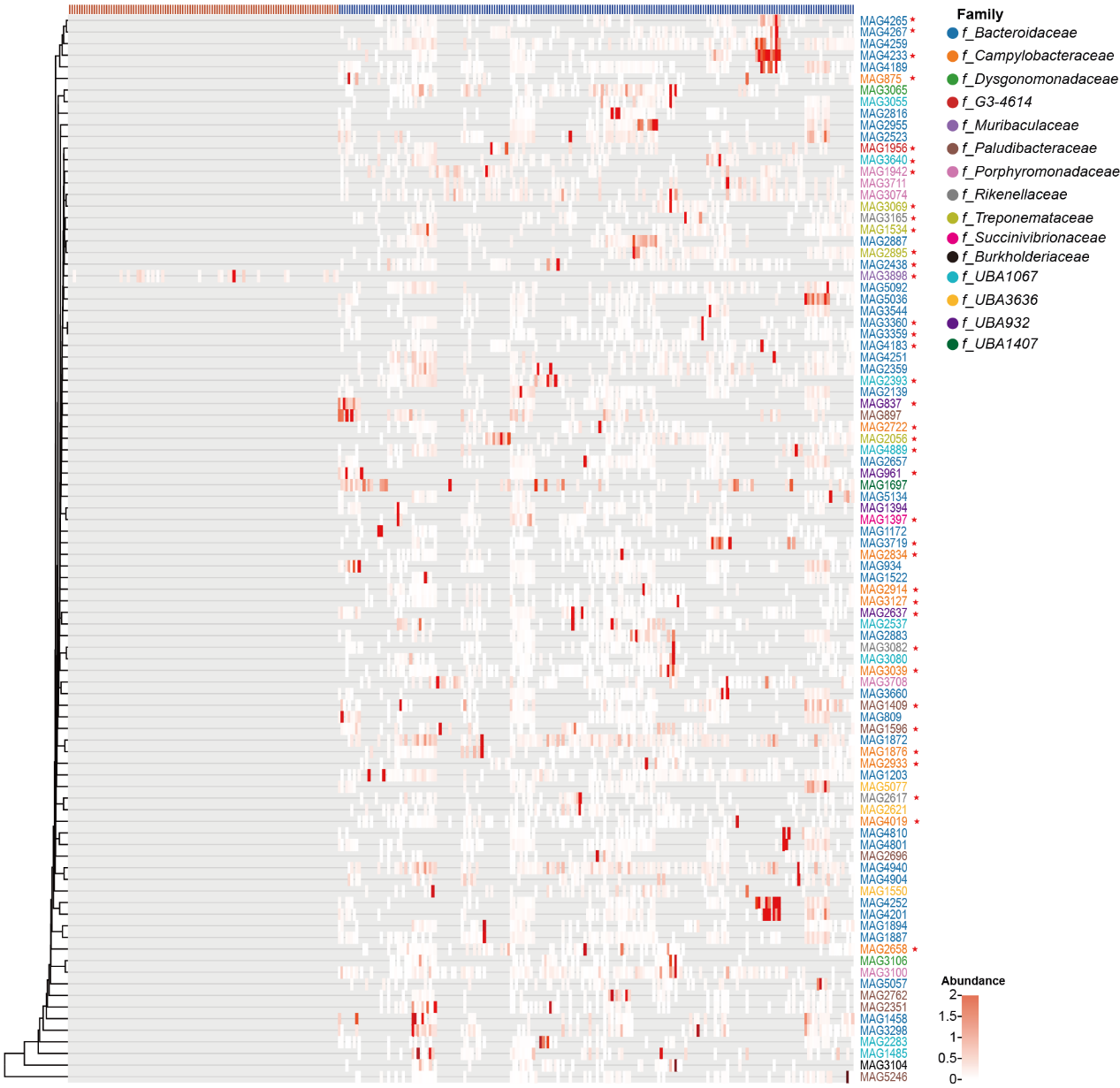
**

**Figure S8. High precision screening of exclusively colonized strains in the gut of sheep and goats based on the MAG level.** Identified 91 MAGs specific to the gut of sheep and one MAG specific to the hindgut of goats. Screening was performed for MAGs that were detected in more than 20% of individuals in either sheep or goats, respectively. Different color MAGs names represent different family levels. MAGs marked with an asterisk represent currently unknown genomes.

**
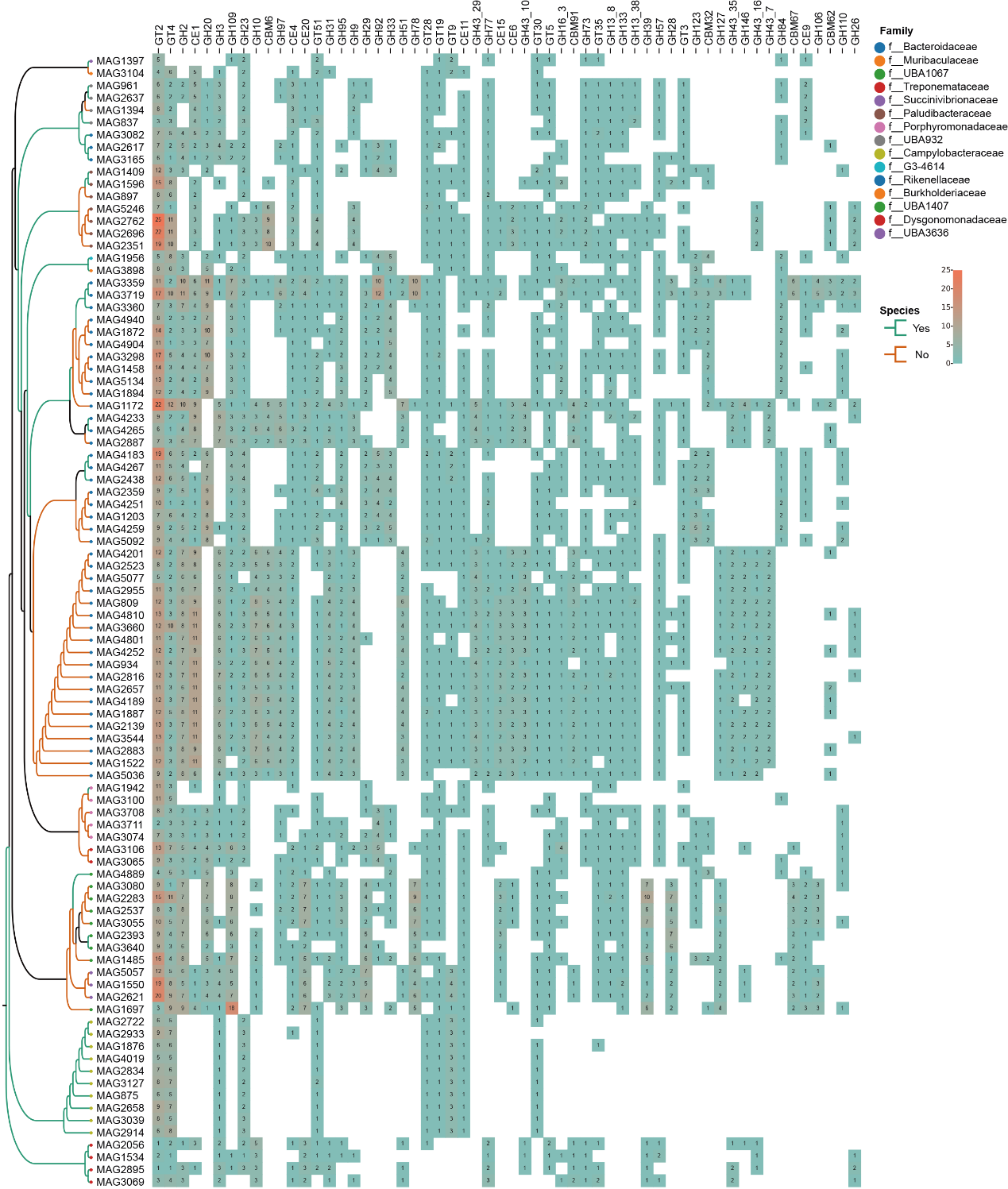
**

**Figure S9. Characteristics exploration of 91 MAGs encoding CAZy genes specifically colonizing in sheep.** The classification of the unique 91 MAGs in the gut of sheep was analyzed. Maximum-likelihood tree of the 91 specifically colonizing in sheep genomes constructed using PhyloPhlAn. The heatmap represents the number of mucin-degrading CAZy enzyme genes encoded by each MAG. Blank spaces indicate that the corresponding enzyme genes were not detected in the genome of these MAGs.
